# Co-occurring genomic alterations and immunotherapy efficacy in NSCLC

**DOI:** 10.1038/s41698-021-00243-7

**Published:** 2022-01-18

**Authors:** Fan Zhang, Jinliang Wang, Yu Xu, Shangli Cai, Tao Li, Guoqiang Wang, Chengcheng Li, Lei Zhao, Yi Hu

**Affiliations:** 1 Department of Oncology and Institute of Translational Medicine, Medical Innovation Research Center and The Fifth Medical Center, Chinese PLA General Hospital, Beijing, China; 2 Medical Department, Burning Rock Biotech, Guangdong, China

**Keywords:** Predictive markers, Cancer immunotherapy, Cancer genetics, Non-small-cell lung cancer

## Abstract

An oncogene-centric molecular classification paradigm in non-small cell lung cancer (NSCLC) has been established. Of note, the heterogeneity within each oncogenic driver-defined subgroup may be captured by co-occurring mutations, which potentially impact response/resistance to immune checkpoint inhibitors (ICIs). We analyzed the data of 1745 NSCLCs and delineated the landscape of interaction effects of common co-mutations on ICI efficacy. Particularly in nonsquamous NSCLC, *KRAS* mutation remarkably interacted with its co-occurring mutations in *TP53*, *STK11*, *PTPRD*, *RBM10*, and *ATM*. Based on single mutation-based prediction models, adding interaction terms (referred to as inter-model) improved discriminative utilities in both training and validation sets. The scores of inter-models exhibited undifferentiated effectiveness regardless of tumor mutational burden and programmed death-ligand 1, and were identified as independent predictors for ICI benefit. Our work provides novel tools for patient selection and insights into NSCLC immunobiology, and highlights the advantage and necessity of considering interactions when developing prediction algorithms for cancer therapeutics.

## Introduction

An oncogene-centric molecular classification paradigm in non-small cell lung cancer (NSCLC) has been established by the discoveries of mutual-exclusive oncogenic drivers, e.g., *KRAS*, *EGFR*, *BRAF*, and *ALK*^1^. However, growing evidence points toward the biological and clinical heterogeneity within each oncogenic driver-defined subgroup^1–6^, which warrants further investigations into the indicators for optimizing the stratification framework in NSCLC.

Co-existing genomic aberrations in oncogenic drivers and tumor suppressor genes have emerged as the main principle of molecular diversity in NSCLC. This co-mutation pattern can capture the complexity concerning tumorigenesis, metastasis, immune microenvironment, and therapeutic vulnerabilities^1,7^. For instance, *KRAS*-driven lung adenocarcinomas (LUADs) are intrinsically heterogeneous and can be classified into three subgroups dominated, respectively, by co-mutations in *TP53*, *STK11*, and *CDKN2A/B*^8^. *KRAS/TP53* co-mutations are associated with an inflamed immune microenvironment and increased tumoral programmed death-ligand 1 (PD-L1) expression^8–11^; However, *KRAS/STK11* co-mutated LUADs appear largely “immune-inert”, characterized by a paucity of tumor-infiltrating T cells and lower PD-L1 expression^8,10–12^. These two different co-occurring mutations in *KRAS*-mutated LUADs lead to almost opposite microenvironments, demonstrating the critical impact of co-occurring mutations on the immune-related characteristics in NSCLC and the promising opportunities that interactions between co-occurring mutations may foster the improvement of prediction algorithms for cancer immunotherapy.

The predictive impacts of two co-occurring mutations may be interactive, but not simply additive, which requires further assessment of interaction effects^13–15^. We previously revealed an interaction of *KRAS*/*TP53* mutations in nonsquamous NSCLC, where we observed similarly poor progression-free survival (PFS) in the patients with no or single mutation, but significant PFS advantage in the co-mutant ones^16^, which indicates that only when these two mutations occur simultaneously, may they predict remarkable benefits from ICIs. In a recently published 8-gene mutational signature, *TP53* and *KRAS* mutations were calculated independently with a score associated with better response to ICIs^17^. In this case, the single-mutated patients had a higher score compared to the double-wildtype ones, inconsistent with their similarly poor outcomes in the actual situation. Co-mutations are rare and therefore assessing interaction effects requires a large sample size to avoid serious sampling error, which partially accounts for the omission of interaction effects in current mutational signatures for predicting immunotherapy efficacy in NSCLC^17,18^.

Given these, we hypothesized that co-occurring mutations may shape immune contexture and act as novel predictors for immunotherapy efficacy in NSCLC. In this study, we sought to first delineate the landscape of interaction effects separately in patients with nonsquamous or squamous NSCLC, and then to investigate whether adding significant interaction terms into prediction models could improve their performances in both the training and the validation sets. Our goal was to develop a novel tool involving interaction terms to predict ICI benefit more precisely in NSCLC and to raise the awareness of involving co-occurring genomic alterations for facilitating the refinement of prediction algorithms for cancer therapeutics.

## Results

### Interaction between mutational events was associated with immunotherapy efficacy

**Fig. 1:**
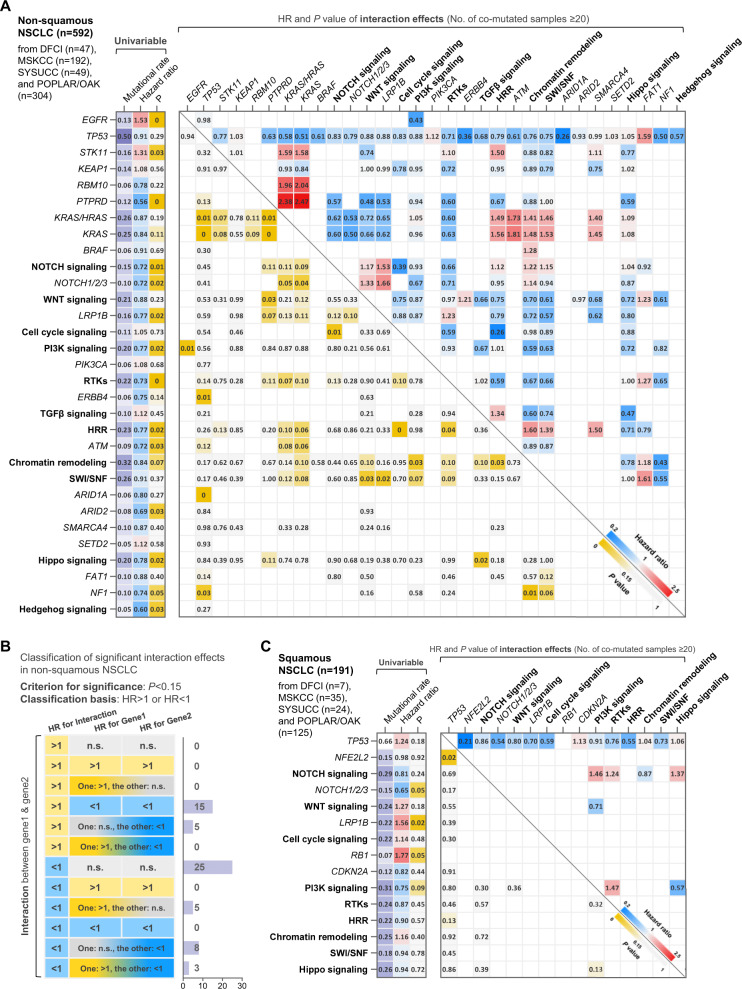
Associations of ICI efficacy with single mutations and interactions of co-occurring mutations in NSCLC. **A** Landscape of the predictive utilities of single mutations (left) and the interaction effects of common co-occurring mutations (right) in nonsquamous NSCLC. **B** Classification of interaction effects based on the effect sizes of single mutation terms and interaction terms. **C** Landscape of the predictive utilities of single mutations (left) and the interaction effects of common co-occurring mutations (right) in squamous NSCLC. Abbreviations: NSCLC nonsmall cell lung cancer.

To begin with, we analyzed whether mutational events in single genes or pathways were associated with the PFS on anti-PD-(L)1 monotherapy in nonsquamous NSCLC (*n* = 592). The detailed information and survival data of included datasets are shown in Supplementary Table 1 and Supplementary Fig. 1, and the definitions of analyzed pathways are shown in Supplementary Table 2. Univariable analyses revealed that poorer PFS on anti-PD-(L)1 monotherapy was associated with the mutations in *EGFR* (*P* = 0.001) and *STK11* (*P* = 0.026), and better PFS was associated with the mutations in *PTPRD* (*P* < 0.001), NOTCH pathway (*P* = 0.009), *NOTCH1/2/3* (*P* = 0.025), *LRP1B* (*P* = 0.025), PI3K pathway (*P* = 0.022), receptor tyrosine kinases (RTKs, *P* = 0.004), *SMAD4* (*P* = 0.046), homologous recombination repair (HRR) pathway (*P* = 0.016), *ATM* (*P* = 0.029), *ATR* (P = 0.049), *ARID2* (*P* = 0.032), Hippo pathway (*P* = 0.023), and Hedgehog pathway (P = 0.032, Fig. 1A and Supplementary Table 3).

As for the interaction effects between co-occurring genetic aberrations, the co-mutations existing in more than 20 patients were included in the analysis to reduce the impact of serious sampling error. We found 20 significant interactions with a *P* value below 0.05, and 41 interactions with a *P* value between 0.05 and 0.15 (Fig. 1A). Among these 61 interactions, 28 (45.9%) had effect sizes opposite to the trends of the effect sizes of single mutations (e.g., PTPRD*KRAS pathway: hazard ratio [HR] = 2.47, 95% confidence interval (CI) 1.33-4.61, *P* = 0.004; *PTPRD*: HR = 0.43, 95% CI 0.30-0.62, *P* < 0.001; *KRAS*: HR = 0.76, 95% CI 0.60-0.95, *P* = 0.018, Fig. 1B and Supplementary Table 4). These results indicate that gene mutations may exhibit different associations with immunotherapy efficacy based on their co-occurring mutations in nonsquamous NSCLC.

In the patients with squamous NSCLC (*n* = 191), the relatively small sample size limits the exploration of significant effects to a certain extent, and therefore we relaxed the requirement for *p* value from 0.05 to 0.15 in the following analysis. In terms of individual gene mutations, the mutations in *NOTCH1/2/3* (*P* = 0.053) and PI3K pathway (*P* = 0.092) were associated with longer PFS on anti-PD-(L)1 monotherapy and the mutations in *LRP1B* (*P* = 0.016) and *RB1* (*P* = 0.051) were associated with worse PFS (Fig. 1C and Supplementary Table 5). For the interaction effects, three remarkable interactions were discovered (TP53*NFE2L2, TP53*HRR pathway, and PI3K pathway*Hippo pathway, Fig. 1C and Supplementary Table 6). Two interactions in squamous NSCLC (TP53*NFE2L2 and TP53*HRR pathway) had effect sizes opposite to the trends of the effect sizes of single mutations (e.g., TP53*NFE2L2: HR = 0.21, 95% CI 0.06–0.74, *P* = 0.016; *TP53*: HR = 1.37, 95% CI 0.97–1.92, *P* = 0.071; *NFE2L2*: HR = 3.71, 95% CI 1.15–12.00, *P* = 0.029), highlighting the importance of investigating interaction effects of co-occurring mutations.

### Development of a model involving interactions for anti-PD-(L)1 therapy in nonsquamous NSCLC

**Fig. 2:**
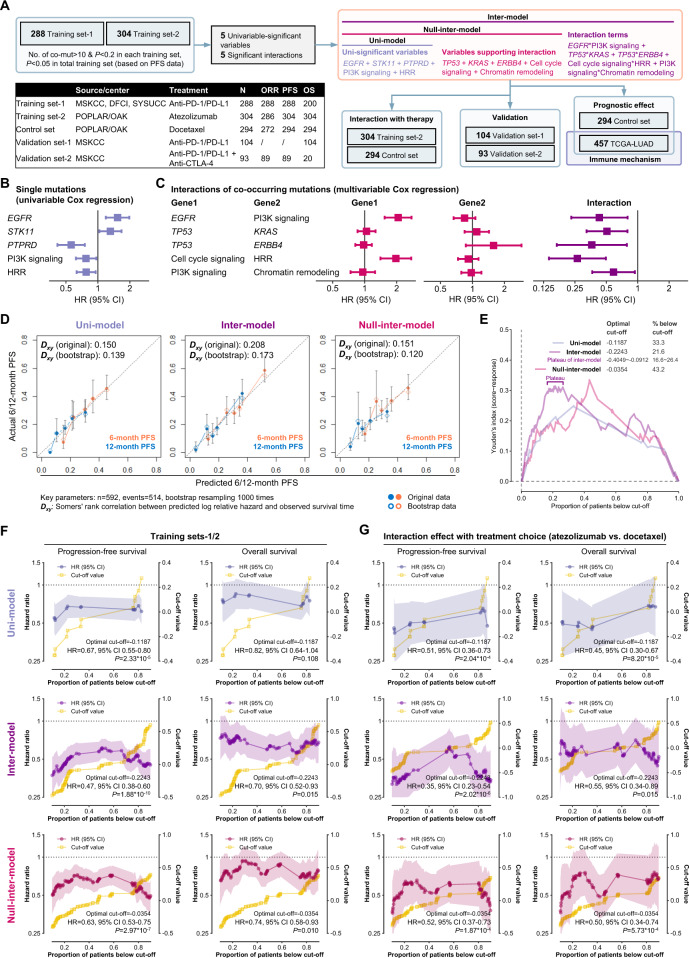
Performances of the three models in the training sets of nonsquamous NSCLC. **A** Workflow of developing and validating three models in nonsquamous NSCLC. **B**, **C** The single mutations (**B**) and interaction effects of co-mutations (**C**) selected for model development. **D** Calibration curves of the three models in the training sets-1/2. **E** Youden’s index based on the receiver operating characteristic curve of the three inter-scores and response to anti-PD-(L)1 monotherapy in the training sets-1/2. **F** Performances of the three models on discriminating the PFS and OS on anti-PD-(L)1 monotherapy in the training sets-1/2. **G** Performances of the three models on predicting the PFS and OS benefit from atezolizumab over docetaxel in the POPLAR/OAK cohort. Abbreviations: DFCI Dana Farber Cancer Institute, MSKCC Memorial Sloan-Kettering Cancer Center, NSCLC nonsmall cell lung cancer, OS overall survival, PD-1 programmed death-1, PD-L1 programmed death-ligand 1, PFS progression-free survival, SYSUCC Sun Yat-Sen University Cancer Center.

As shown in Fig. 2A, of the total 1083 patients with nonsquamous NSCLC, 288 anti-programmed death-(ligand)1 (anti-PD-(L)1) monotherapy-treated patients with PFS data from Sun Yat-Sen University Cancer Center (SYSUCC)^19^, Dana Farber Cancer Institute (DFCI)^20^, and Memorial Sloan-Kettering Cancer Center (MSKCC)^21–23^ were included in the training set-1, and 304 patients treated with atezolizumab and 294 patients treated with docetaxel from the POPLAR/OAK cohort were included in the training set-2 and the control set, respectively^24^.

By cross-validation in training sets-1/2 (detailed criteria were shown in Fig. 2A: [i] number of mutation or co-mutation>10 and *P* < 0.2 in training set-1 [*n* = 288] and -2 [*n* = 304]; [ii] *P* < 0.05 in total training sets [*n* = 592]), five single mutational events (*EGFR*, *STK11*, *PTPRD*, PI3K pathway, and HRR pathway, Fig. 2B), and five interactions (EGFR*PI3K pathway, TP53*KRAS, TP53*ERBB4, cell cycle pathway*HRR pathway, and PI3K pathway*chromatin remodeling pathway, Fig. 2C) were selected to develop three different prediction models. The first model only includes the five single mutational events (termed uni-model); The second model consists of the five single mutations, five interactions, and five terms that support the valid calculation of interaction effects (termed inter-model); The third model was designed to be a control model between the uni-model and the inter-model, contains five single mutations and five supporting terms, but no interaction terms (termed null-inter-model, Fig. 2A). By comparing the performances of these three models (coefficients: Supplementary Table 8, nomograms: Supplementary Fig. 2), it was possible to evaluate whether adding interaction terms into the prediction model with only individual mutations could improve its predictive effectiveness.

In the training sets-1/2, lower scores of the inter-model were associated with better PFS, and the inter-model showed numerically better discriminative performance (bootstrap *D*_*xy*_ = 0.173) and predictive utility for the response to anti-PD-(L)1 monotherapy (area under the curve of receiver operating characteristic [AUROC] = 0.690 [*P* = 7.0*10^−^]) compared to the uni-model (bootstrap *D*_*xy*_ = 0.139, AUROC = 0.655 [*P* = 4.7*10^−7^]) and the null-inter-model (bootstrap *D*_*xy*_ = 0.120, AUROC = 0.673 [*P* = 2.0*10^−8^], Fig. 2D–E and Supplementary Fig. 3A). As genomic features likewise, the AUROCs of tissue tumor mutational burden (tTMB, 0.603, *P* = 0.026) and blood TMB (bTMB, 0.547, *P* = 0.445) were lower than all three models (Supplementary Fig. 3A). The potentially optimal cut-off of each model was identified when Youden’s index reached the maximum (Fig. 2E). In addition to the optimal cut-off, other cut-off values were also taken into consideration. We compare the survival data of the patients with a score below each cut-off with those with a score above this cut-off. By this methodology, we aimed to comprehensively evaluate the robustness of predictive effectiveness for each model.

Lower scores were associated with longer survival on anti-PD-1/PD-L1 monotherapy (Fig. 2F), but poorer survival on docetaxel and prognosis in The Cancer Genomic Atlas (TCGA)-LUAD cohort (Supplementary Fig. 4). By directly calculating the interaction effect between treatment (atezolizumab vs. docetaxel) and each score in the POPLAR/OAK cohort, all three models exhibited excellent discriminative effectiveness in predicting PFS benefit from atezolizumab over docetaxel. However, when it comes to predicting OS benefit, only the uni-model and the inter-model showed good discriminative effectiveness (Fig. 2G), indicating their predictive utility.

### Outperformance and robustness of the inter-model in predicting immunotherapy efficacy in nonsquamous NSCLC

**Fig. 3:**
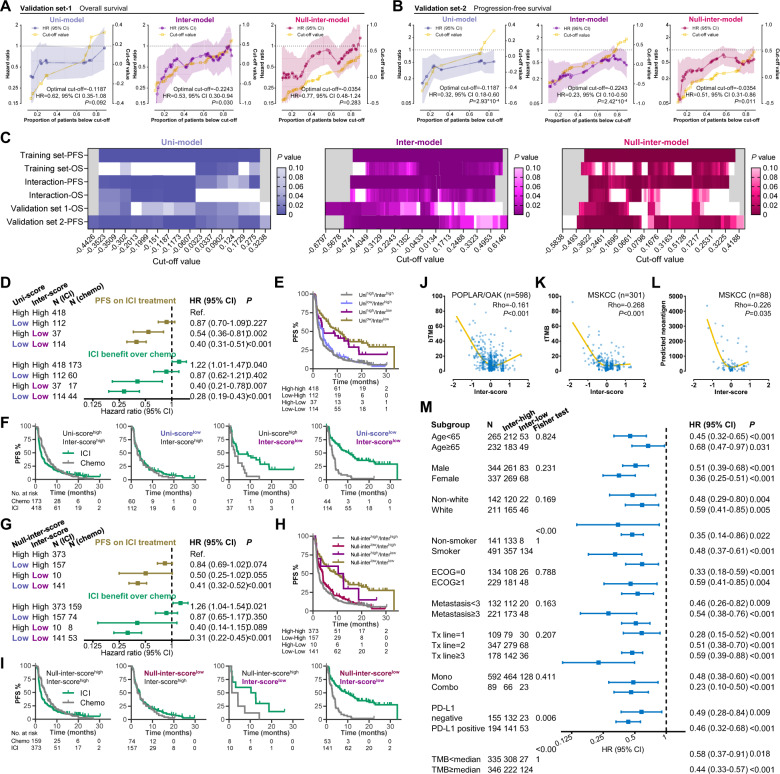
Validation and comparison of the three models and potential immune-related mechanism of the inter-score in nonsquamous NSCLC. **A**, **B** Performances of the three models on discriminating the OS on anti-PD-(L)1 monotherapy in the validation set-1 (**A**) and the PFS on combination therapy with anti-CTLA-4 in the validation set-2 (**B**). **C** Summary of the performances of the three models in all training/validation sets. **D–F** Comparison between the uni-model and the inter-model when two results are inconsistent (**D**). KM curves illustrating PFS on ICI treatment (**E**) and PFS benefit from ICI therapy over docetaxel (**F**). **G–I** Comparison between the null-inter-model and the inter-model when two results are inconsistent (**G**). KM curves illustrating PFS on ICI treatment (**H**) and PFS benefit from ICI therapy over docetaxel (**I**). **J–L** Association of the inter-score with blood TMB (**J**), tissue TMB (**K**), and neoantigen load (**L**). **M** Effectiveness of the inter-score in patients with different clinicopathological features, TMB, and PD-L1. Abbreviations: CTLA-4 cytotoxic T lymphocyte antigen-4, ICI immune checkpoint inhibitor, KM Kaplan-Meier, NSCLC nonsmall cell lung cancer, OS overall survival, PD-1 programmed death-1, PD-L1 programmed death-ligand 1, PFS progression-free survival, TMB tumor mutational burden.

First, two cohorts were employed to validate the models for immunotherapy in nonsquamous NSCLC. As mentioned in Fig. 2A, the validation set-1 consists of 104 patients with only OS data, who had received anti-PD-(L)1 monotherapy, and the validation set-2 includes 93 patients with objective response rate (ORR) and PFS data treated with combination therapy with anti-cytotoxic T lymphocyte antigen-4 (anti-CTLA-4). In the validation set-1, the inter-model outperformed the other two models on account of (1) wider range of applicable cut-off values and (2) the significant result at optimal cut-off (Fig. 3A). In the validation set-2, all three models showed good discriminative effectiveness. However, compared to the other two models, the inter-model exhibited a numerically higher AUROC of response (inter-model: 0.816, *P* = 2.0*10^−6^; uni-model: 0.692, *P* = 0.003; null-inter-model: 0.708, *P* = 0.002, Supplementary Fig. 3B), and a lower HR value at optimal cut-off (Fig. 3B).

Second, we sought to compare the utility of three models by summarizing the *P* values at all cut-offs in both training and validation sets (Fig. 3C). The inter-score exhibited consistent discriminative effectiveness ranging from −0.4059 to 0.0163 (approximately from 15th percentile to 55th percentile), outperforming the other two scores. To further compare the inter-model with the other two models, we classified all patients with nonsquamous NSCLC by the optimal cut-offs and compared their survival outcomes (all HR and 95% CI results below are summarized in Fig. 3D). To start with, we compared the inter-model with the uni-model. As shown in Fig. 3E illustrating the PFS on ICI treatment, the uni-score^high^/inter-score^high^ subgroup had the shortest PFS. Compared to this subgroup, slightly longer PFS was observed in the uni-score^low^/inter-score^high^ subgroup (HR = 0.87, 95% CI 0.70–1.29, *P* = 0.227), but markedly longer PFS was revealed in the uni-score^high^/inter-score^low^ subgroup (HR = 0.54, 95% CI 0.36–0.81, *P* = 0.002). As shown in Fig. 3F, compared to chemotherapy, ICI treatment decreased the hazard of progression or death by only 13% in the uni-score^low^/inter-score^high^ subgroup (HR = 0.87, 95% CI 0.62–1.21, *P* = 0.402), and by higher proportion as 60% in the uni-score^high^/inter-score^low^ subgroup (HR = 0.40, 95% CI 0.21–0.78, *P* = 0.007). These results demonstrate that when the results of the two models are inconsistent, the inter-model may be more accurate in predicting ICI benefit. We further compared the inter-model with the null-inter-model, and similar superiority of the inter-model was observed (Fig. 3G–I). Taken together, the inter-model involving interaction effects of co-occurring genomic alterations outperformed the other two control models, indicating the necessity of adding interaction terms into the prediction model for optimizing its discriminative utility.

Third, we proposed to examine the applicability of the inter-model in the nonsquamous NSCLCs with different clinical characteristics. As for TMB and PD-L1, lower inter-score was weakly associated with higher TMB and neoantigen load (Rho < 0.30, Fig. 3J–L) and PD-L1 positivity (69.7% vs. 51.6%, *P* = 0.006). However, consistent predictive effectiveness was revealed in the TMB < median (HR = 0.58, 95% CI 0.37–0.91, *P* = 0.018), the TMB ≥ median (HR = 0.44, 95% CI 0.33–0.57, *P* < 0.001), the PD-L1^negative^ (HR = 0.49, 95% CI 0.28–0.84, *P* = 0.009), and the PD-L1^positive^ nonsquamous NSCLCs (HR = 0.46, 95% CI 0.32–0.68, *P* < 0.001, Fig. 3M). Moreover, the inter-score showed undifferentiated predictive value regardless of other key clinicopathological features (age, sex, race, smoking, Eastern Cooperative Oncology Group [ECOG], number of metastasis sites, treatment lines, and ICI regimen, Fig. 3M). These results demonstrate the robustness of the inter-model in nonsquamous NSCLC with different clinical characteristics.

### Immune-related mechanisms of the inter-score in nonsquamous NSCLC

**Fig. 4:**
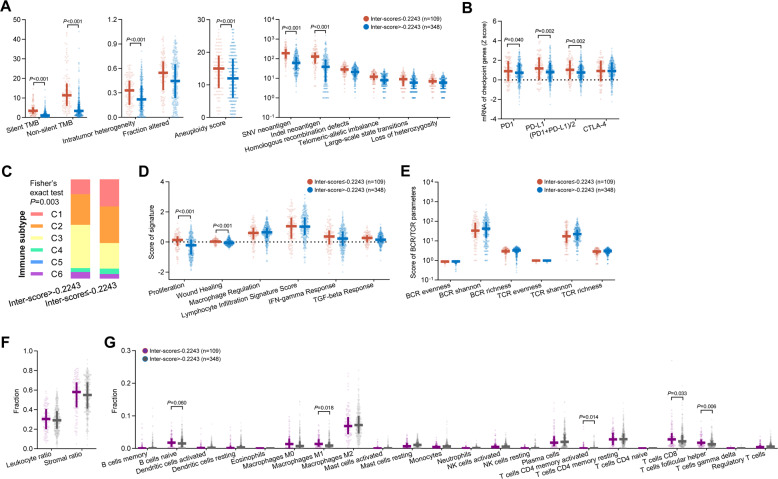
Immune correlates of the inter-score in the TCGA-LUAD cohort. **A**–**G** Associations of the inter-score with TMB, intratumor heterogeneity, fraction altered, aneuploidy score, neoantigen load, homologous recombination defects (**A**), mRNA of immune checkpoint genes (**B**), immune subtype (**C**), signatures supporting immune subtyping (**D**), and BCR/TCR parameters (**E**), leukocyte/stromal ratio (**F**), and tumor-infiltrating immune cells (**G**). Abbreviations: BCR B cell receptor, LUAD lung adenocarcinoma, SNV single-nucleotide variation, TCGA The Cancer Genomic Atlas, TCR T cell receptor, TMB tumor mutational burden.

The TCGA-LUAD cohort with genomic, transcriptomic, and survival data was used for exploring the immune-related mechanisms of the inter-score. Similar to the previous result about TMB and PD-L1 expression, higher TMB, neoantigen load, aneuploidy score, intratumoral heterogeneity, PD-1 mRNA, and PD-L1 mRNA were observed in the inter-score^low^ group (Fig. 4A, B).

Thorsson et al. presented immunogenomics analyses of more than 10,000 tumors in TCGA, identifying 6 immune subtypes (C1: wound healing, C2: IFN-γ dominant, C3: inflammatory, C4: lymphocyte depleted, C5: immunologically quiet, C6: TGF-β dominant) that encompass 33 cancer types based on six key signatures^25^. Higher prevalence of C1 and C2 and lower prevalence of C3 were observed in the inter-score^low^ group (*P* = 0.003, Fig. 4C, D). Both C1 and C2 subtypes had low Th1/Th2 ratio, high proliferation rate, high intratumoral heterogeneity, and C2 had the highest M1/M2 macrophage polarization, and a strong CD8 signal^25^. In addition, no significant difference of the features of B and T cell receptors (BCR/TCR) were observed (Fig. 4E). Regarding immune cell infiltration, despite the similar fractions of leukocyte and stromal area in both groups (Fig. 4F), greater infiltration of naïve B cell (*P* = 0.060), M1 macrophage (*P* = 0.018), activated memory CD4^+^ T cell (*P* = 0.014), CD8^+^ T cell (*P* = 0.033), and follicular helper T cell (*P* = 0.006) was found in the inter-score^low^ group (Fig. 4G).

These immune-related associations in addition to TMB and PD-L1 may serve as the foundation explaining why its predictive utility for immunotherapy efficacy was undifferentiated in the patients with different levels of TMB and PD-L1 expression (Fig. 3M). To further confirm this independence, we performed multivariable analysis including the inter-score, TMB, PD-L1, and key clinicopathological features in the patients with valid TMB and PD-L1 data (Table 1). Lower inter-score, rather than high TMB and PD-L1 positivity, was independently associated with longer PFS on ICI treatment (inter-score: multivariable HR = 0.45, 95% CI 0.31–0.67, *P* < 0.001) rather than docetaxel (multivariable HR = 1.16, 95% CI 0.80–1.68, *P* = 0.425). More importantly, the interaction effect between the inter-score and treatment choice (ICI vs. chemotherapy) was remarkably significant (multivariable HR = 0.38, 95% CI = 0.23–0.61, *P* < 0.001). These findings demonstrate that the inter-score, rather than TMB and PD-L1, may be a robust and independent predictor for favorable benefit from ICIs in nonsquamous NSCLC.

**Table 1:** Univariable and multivariable analyses of PFS in patients with nonsquamous NSCLC or squamous NSCLC.

Nonsquamous NSCLC	ICI treatment (*n* = 349)				Chemotherapy (*n* = 224)				Interaction effect (*n* = 573)			
Parameter	Univariable analysis		Multivariable analysis		Univariable analysis		Multivariable analysis		Univariable analysis		Multivariable analysis	
	HR (95%CI)	*P* value	HR (95%CI)	*P* value	HR (95%CI)	*P* value	HR (95%CI)	*P* value	HR (95%CI)	*P* value	HR (95%CI)	*P* value
Age (≥65 vs. <65)	1.06 (0.81–1.39)	0.659			0.94 (0.72–1.24)	0.680			1.00 (0.82–1.21)	0.970		
Sex (male vs. female)	0.81 (0.64–1.02)	0.069	0.79 (0.58–1.08)	0.144	1.02 (0.78–1.35)	0.872			0.88 (0.74–1.05)	0.169		
Race (white vs. nonwhite)	0.81 (0.62–1.04)	0.099	1.02 (0.75–1.39)	0.900	0.99 (0.74–1.33)	0.954			0.88 (0.72–1.06)	0.184		
Smoking (smoker vs. non-smoker)	0.72 (0.55–0.95)	0.022	0.80 (0.55–1.16)	0.245	1.00 (0.71–1.42)	0.984			0.80 (0.64–0.99)	0.040	0.83 (0.65–1.06)	0.142
ECOG (≥1 vs. 0)	1.21 (0.94–1.56)	0.145			1.40 (1.05–1.86)	0.021	1.35 (1.01–1.80)	0.040	1.26 (1.04–1.53)	0.016	1.49 (1.21–1.84)	<0.001
Metastatic sites (≥3 vs. <3)	1.51 (1.16–1.97)	0.002	1.61 (1.19–2.17)	0.002	1.55 (1.15–2.08)	0.004	1.40 (1.04–1.89)	0.027	1.49 (1.23–1.81)	<0.001	1.49 (1.21–1.84)	<0.001
Treatment lines (≥3 vs. 2 vs. 1)	1.25 (1.06–1.47)	0.007	0.73 (0.53–1.00)	0.048	0.72 (0.53–0.98)	0.036	0.72 (0.53–0.98)	0.037	1.15 (1.01–1.32)	0.035	0.70 (0.56–0.87)	0.001
PD-L1 (positive vs. negative)	0.73 (0.58–0.92)	0.007	0.82 (0.61–1.09)	0.173	0.93 (0.71–1.23)	0.624	0.94 (0.71–1.24)	0.660	0.79 (0.67–0.95)	0.010	0.89 (0.73–1.08)	0.240
TMB ( ≥ median vs. <median)	0.79 (0.63–1.00)	0.046	1.56 (1.14–2.13)	0.005	1.74 (1.32–2.31)	<0.001	1.59 (1.17–2.17)	0.003	1.00 (0.84-1.19)	0.977	1.57 (1.25-1.96)	<0.001
Inter-score (≤cut-off vs. >cut-off)	0.45 (0.33–0.61)	<0.001	0.45 (0.30–0.67)	<0.001	1.42 (1.02–1.98)	0.039	1.16 (0.80–1.68)	0.425	1.35 (0.97–1.88)	0.077	1.13 (0.80–1.59)	0.494
Therapy (ICI vs. chemotherapy)									1.08 (0.89–1.32)	0.440	1.17 (0.94–1.46)	0.158
Interaction: inter-score*therapy									0.31 (0.20–0.49)	<0.001	0.38 (0.23–0.61)	<0.001

Squamous NSCLC	ICI treatment (*n* = 135)				Chemotherapy (*n* = 91)				Interaction effect (*n* = 226)			
Parameter	Univariable analysis		Multivariable analysis		Univariable analysis		Multivariable analysis		Univariable analysis		Multivariable analysis	
	HR (95%CI)	*P* value	HR (95%CI)	*P* value	HR (95%CI)	*P* value	HR (95%CI)	*P* value	HR (95%CI)	*P* value	HR (95%CI)	*P* value
Age (≥65 vs. <65)	0.88 (0.57–1.36)	0.570			1.63 (1.05–2.53)	0.028	1.56 (1.00–2.42)	0.049	1.12 (0.83–1.51)	0.455		
Sex (male vs. female)	0.79 (0.46–1.36)	0.398			1.40 (0.84–2.32)	0.195			1.03 (0.72–1.49)	0.861		
Race (white vs. nonwhite)	1.00 (0.67–1.50)	0.981			1.32 (0.79–2.21)	0.283			1.16 (0.85–1.57)	0.349		
Smoking (smoker vs. non-smoker)	0.91 (0.44–1.89)	0.805			0.66 (0.16-2.73)	0.568			0.96 (0.51-1.83)	0.908		
ECOG (≥1 vs. 0)	1.40 (0.87–2.26)	0.166			1.26 (0.78–2.02)	0.344			1.28 (0.92–1.79)	0.145		
Metastatic sites (≥3 vs. <3)	1.30 (0.88–1.93)	0.193			0.75 (0.48–1.15)	0.185			1.11 (0.83–1.48)	0.484		
Treatment lines (≥3 vs. 2 vs. 1)	1.41 (1.06–1.87)	0.019	1.53 (1.16–2.03)	0.003	0.88 (0.46-1.67)	0.697			1.35 (1.06–1.72)	0.016	1.40 (1.09–1.78)	0.007
PD-L1 (positive vs. negative)	0.99 (0.67–1.45)	0.954	1.11 (0.75–1.64)	0.599	1.27 (0.82–1.95)	0.280	1.17 (0.76–1.80)	0.483	1.04 (0.79–1.38)	0.775	1.15 (0.87–1.54)	0.328
TMB ( ≥ median vs. <median)	0.83 (0.57–1.21)	0.340	0.69 (0.47–1.01)	0.054	1.58 (1.02–2.44)	0.040	1.52 (0.98–2.36)	0.064	1.00 (0.76–1.32)	1.000	0.97 (0.73–1.29)	0.815
Inter-score (≤cut-off vs. >cut-off)	0.43 (0.29–0.64)	<0.001	0.37 (0.24–0.56)	<0.001	1.12 (0.71–1.78)	0.616	1.14 (0.72–1.81)	0.565	1.08 (0.69–1.70)	0.741	1.07 (0.68-1.68)	0.779
Therapy (ICI vs. chemotherapy)									1.03 (0.73–1.46)	0.850	1.09 (0.77–1.56)	0.619
Interaction: inter-score*therapy									0.37 (0.20–0.69)	0.001	0.35 (0.19–0.65)	<0.001

*CI* confidence interval, *ECOG* Eastern Cooperative Oncology Group, *HR* hazard ratio, *PD-L1* programmed death-ligand 1, *TMB* tumor mutational burden.

### Involving interaction effects in prediction model improved predictive utility in squamous NSCLC

**Fig. 5:**
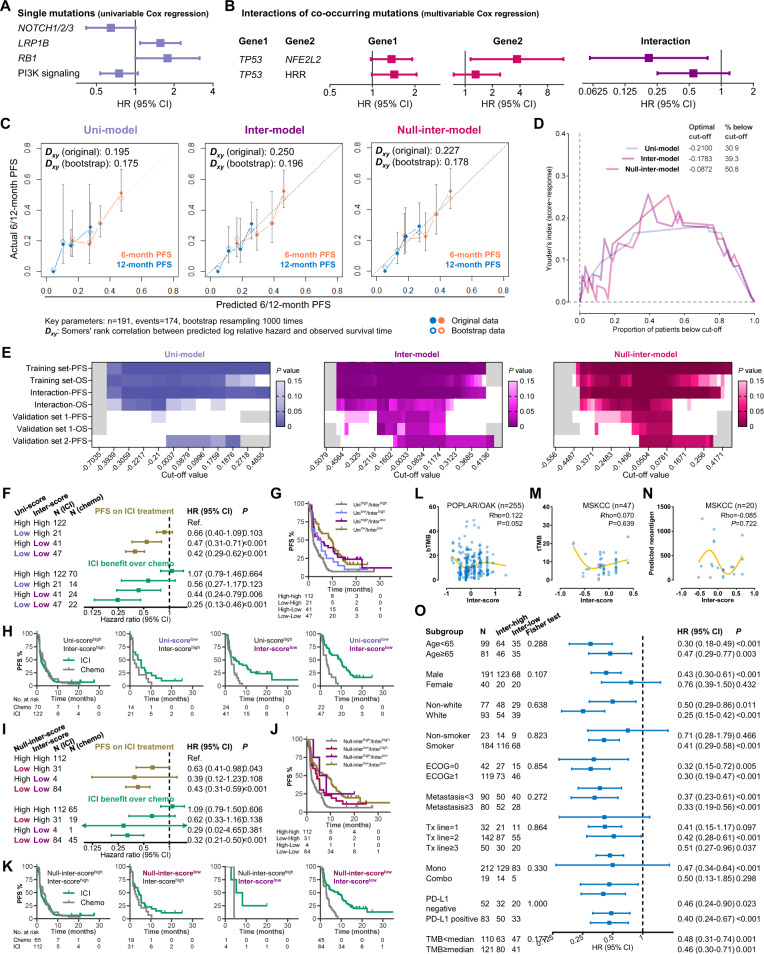
Outperformance of the inter-model in squamous NSCLC. **A**, **B** The single mutations (**A**) and interaction effects of co-mutations (**B**) selected for model development. **C** Calibration curves of the three-models in the training sets-1/2. **D** Youden’s index based on the receiver operating characteristic curve of the three inter-scores and response to anti-PD-(L)1 monotherapy in the training sets-1/2. **E** Summary of the performances of the three models in all training/validation sets. **F–H** Comparison between the uni-model and the inter-model when two results are inconsistent (**F**). KM curves illustrating PFS on ICI treatment (**G**) and PFS benefit from ICI therapy over docetaxel (**H**). **I–K** Comparison between the null-inter-model and the inter-model when two results are inconsistent (**I**). KM curves illustrating PFS on ICI treatment (**J**) and PFS benefit from ICI therapy over docetaxel (**K**). **L–N** Association of the inter-score with blood TMB (**L**), tissue TMB (**M**), and neoantigen load (**N**). **O** Effectiveness of the inter-score in patients with different clinicopathological features, TMB, and PD-L1. Abbreviations: ICI immune checkpoint inhibitor, KM Kaplan-Meier, NSCLC nonsmall cell lung cancer, OS overall survival, PD-1 programmed death-1, PD-L1 programmed death-ligand 1, PFS progression-free survival, TMB tumor mutational burden.

A refinement of the prediction model was observed in nonsquamous NSCLC by considering the interaction effects of co-occurring mutations. We hereby performed the same analyses in squamous NSCLC to investigate whether this improvement could occur in squamous NSCLC as well. To avoid repetition, we shall briefly describe the results. The available data of the training sets and the validation sets are shown in Supplementary Fig. 5. Four single mutational events (mutations in *NOTCH1/2/3*, *LRP1B*, *RB1*, and PI3K pathway) and two interactions (TP53*NFE2L2 and TP53*HRR pathway) were involved for developing the three models (Fig. 5A, B and Supplementary Table 9). The coefficients and the nomograms of these three models are illustrated in Supplementary Table 10 and Supplementary Fig. 6.

In the training sets-1/2, the inter-model showed numerically better discriminative performance (Fig. 5C) and predictive utility on response to anti-PD-(L)1 (Fig. 5D and Supplementary Fig. 7A), PFS/OS on anti-PD-(L)1 (Supplementary Fig. 8A), and survival benefit from atezolizumab over docetaxel (Supplementary Fig. 8B). Similar advantages were seen in validation sets-1/2 (response: Supplementary Fig. 7B-C, PFS/OS: Supplementary Fig. 9). P values at all cut-offs of the analyses in the training sets and the validation sets were summarized in Fig. 5E. The inter-score exhibited consistent discriminative utility (criterion: *P* < 0.15) ranging from −0.1528 to 0.1312 (approximately from 41st percentile to 61st percentile), outperforming the other two scores (uni-model: none; null-inter-model: from −0.0709 [50^th^ percentile] to 0.1094 [61^st^ percentile]). In addition, lower scores were associated with better immunotherapy efficacy rather than longer survival on docetaxel and better prognosis in the TCGA-lung squamous carcinoma (LUSC) cohort (Supplementary Fig. 10), indicating their predictive, but not prognostic utilities.

When the result of the inter-score was opposite to the one of the uni-score (Fig. 5F–H) or the null-inter-score (Fig. 5I–K), the inter-model performed numerically better than the other two models in predicting the PFS on ICI treatment and the PFS benefit from immunotherapy over chemotherapy. These findings demonstrate the necessity of adding interaction terms into the prediction model for optimizing its discriminative utility.

No significant correlation of the inter-score with mutational burden, neoantigen load, and PD-L1 positivity was observed (Fig. 5L–O and Supplementary Fig. 11A–B). As shown in Supplementary Fig. 11, the immune mechanism of the inter-score may include higher mRNA expressions of PD-1 and CTLA-4, BCR richness and Shannon index, the fraction of stromal area and infiltration of naïve B cell, monocyte, M1 macrophage, activated NK cell, resting memory CD4^+^ T cell, and follicular helper T cell. These may be part of the mechanisms underlying its predictive function for ICI treatment and explain why the inter-score showed undifferentiated predictive utility in the patients with different levels of TMB and PD-L1 expression (Fig. 5O).

The inter-score showed concordant associations with PFS in the patients with different TMB, PD-L1, and clinical characteristics, indicating wide applicability. We further performed multivariable analyses (including TMB, PD-L1, and clinical characteristics) and identified lower inter-score as an independent indicator of longer PFS on ICI treatment (inter-score: multivariable HR = 0.37, 95% CI 0.24–0.56, *P* < 0.001) rather than chemotherapy (multivariable HR = 1.14, 95% CI 0.72–1.81, *P* = 0.565), and larger PFS benefit from ICI treatment to chemotherapy (multivariable HR = 0.35, 95% CI = 0.19–0.65, *P* < 0.001).

Altogether, the outperformance of the inter-model over the other two models in squamous NSCLC demonstrates the improvement by adding interaction terms into prediction models. Moreover, good performances in subgroup analysis and multivariable analysis indicate the effectiveness and robustness of the inter-model in predicting immunotherapy efficacy in squamous NSCLC.

## Discussion

In this retrospective study involving 1745 patients from eight cohorts, we delineated the landscape of interaction effects between co-occurring mutations on ICI efficacy and we further developed and validated two cost-effective mutational signatures involving interaction terms to predict ICI benefit more precisely in nonsquamous and squamous NSCLC. Taken together, our comprehensive analysis demonstrates the advantage and necessity of involving interaction effects when developing a prediction algorithm.

Despite abundant studies exploring the mutational biomarkers for ICI treatment, most of these focused on the impact of a single gene (e.g., *EGFR*) or a group of genes with similar functions (e.g., *NOTCH1/2/3* and *EPHA* receptors)^19,20,23,26–28^. Unlike previous studies in nonsquamous NSCLC^29^, *KEAP1* mutation was not associated with poorer ICI efficacy in the training datasets and thereby was not included in the prediction models. Given the inconsistent predictive effects of *KEAP1* mutation in different cohorts (Supplementary Fig. 12A), further investigations of the association between KEAP1 and ICI efficacy are warranted. In the studies aiming at single genes, whether their predictive utilities are influenced by other mutations was not investigated, partially due to the small sample size of co-mutated tumors which could lead to serious sampling error. We hereby collected the PFS data of nearly 800 NSCLC patients treated with anti-PD-(L)1 monotherapy and evaluated the interaction effects with more than 20 co-mutated samples separately in nonsquamous and squamous NSCLC.

Within *KRAS*-mutant LUADs, previous studies have extensively explored the impact of co-occurring mutations (e.g., *TP53*, *STK11*, *CDKN2A/B*) on pathogenesis. In terms of immune-related features, *KRAS/TP53* co-mutated LUADs exhibit high TMB, *CD8A* mRNA, and PD-L1 protein expression^8,9^, while *KRAS/STK11* co-mutations are associated with PD-L1 negativity and T cell suppressive properties, despite an intermediate-to-high TMB^12^. In keeping with their prominent role in shaping immune-related characteristics, co-occurring mutations further impacted survival outcome with ICIs in the present study (KRAS*TP53, HR = 0.51, *P* = 0.003; KRAS*STK11, HR = 1.58, *P* = 0.082). In addition to *TP53* and *STK11* mutations, we also revealed remarkable interactions of *KRAS* mutations with co-occurring genomic alterations in *PTPRD* (HR = 2.47, *P* = 0.004), *RBM10* (HR = 2.04, *P* = 0.094), *NOTCH1/2/3* (HR = 0.50, *P* = 0.036), *ATM* (HR = 1.81, *P* = 0.062), RTKs (HR = 0.63, *P* = 0.097), and switch/sucrose non-fermentable complex (SWI/SNF, HR = 1.53, *P* = 0.079). The immune-related mechanisms of these co-mutations are largely unknown, and our results provide new perspectives for the basic research into tumor immunobiology and immune subtyping in *KRAS*-driven LUADs.

Of note, in a single-center retrospective analysis, the NSCLC patients with *STK11*/*KRAS* co-mutations (*n* = 36) exhibited longer OS on ICI treatment compared to the ones with *STK11* mutation only (*n* = 37)^30^, inconsistent with the previous and our results^12^. The reason for this discord may include that the *STK11*/KRAS co-mutated patients in their cohort were older at diagnosis, more likely to have received nivolumab, and more likely to have longer smoking histories, compared with the *STK11*^mut^/*KRAS*^WT^ patients^30^. This highlights the importance of balancing key clinical characteristics before investigating biomarkers, especially ICI usage and treatment lines.

Among LUSCs, both *TP53* and *NFE2F2* mutations are frequent^31^. A recent study suggested the potential relationship between them by uncovering an NFE2L2-mediated increase in tumor growth seen in *Keap1/Tp53*-double-deleted LUSC cells^32^. We observed a substantial interaction effect between these two mutations impacting the PFS on anti-PD-(L)1 monotherapy (HR = 0.21, *P* = 0.016), which introduces a possibility of the interactive effect of *TP53/NFE2L2* co-mutations on immune phenotypes in LUSC.

The advantage of adding key interaction terms into prediction models to improve discriminative effectiveness in both training and validation sets was revealed by comparing three models (uni-model, inter-model, and null-inter-model) in nonsquamous and squamous NSCLC respectively. In the combined analysis, we further assessed the benefit from ICIs over docetaxel when the results of different models are inconsistent, and the inter-model exhibited better accuracy in predicting ICI benefit, compared to the other two models involving no interaction terms.

Importantly, our inter-models held great promise by their broad applicability, for the equivalent predictive value for ICI treatment regardless of age, sex, race, smoking history, ECOG, metastasis, treatment lines, combination with anti-CTLA-4, TMB level, and PD-L1 expression. Even in the patients with high tissue TMB (≥10) or tumoral PD-L1 ( ≥ 50%), the inter-score can also successfully discriminate the survival outcome with ICI therapy (Supplementary Fig. 13). TMB is not a validated predictive biomarker of survival benefit to ICIs in NSCLC, lacking unified cut-off in several analyses and not predicting survival benefit in some large phase 3 trials (e.g., CheckMate 227 and KEYNOTE-189)^24,33–36^. As genomic features likewise, the inter-model exhibited higher AUROC in predicting response to anti-PD-(L)1 therapy in nonsquamous and squamous NSCLCs. Furthermore, we also noticed that the inter-score was able to identify responders with TMB-low or PD-L1-negative NSCLC, making it a meaningful work for patient selection. The inter-score rather than TMB and PD-L1 is an independent predictor in our multivariable analysis, indicating that the inter-score might in some way cover TMB and PD-L1 with more predictive power. This speculation is partly supported by the association between the inter-score and the immune-related features other than TMB/PD-L1 (e.g., BCR/TCR diversity, mRNA expression of immune checkpoints, and quantity of tumor-infiltrating immune cells). In addition, we assessed the performance at other cut-off values besides the optimal one derived from the ORR-based receiver operating characteristic (ROC) curve (nonsquamous: 15th–55th percentile; squamous: 41st–61st percentile), indicating the robustness of our inter-models.

As for limitation, first, *KRAS*^G12C^ mutations are not reported in the OAK/POPLAR cohort. In the present study, the *TP53*/*KRAS* co-mutation was associated with better immunotherapy efficacy in nonsquamous NSCLCs, and the interaction term was included in the inter-model. In the datasets other than the POPLAR/OAK cohort, we analyzed the PFS on ICIs of the nonsquamous NSCLC patients with *TP53*/*KRAS*^G12C^ co-mutation, which was relatively longer than the ones without co-mutation (*P* = 0.067, Supplementary Fig. 12B). This result indicates that the predictive effect of *KRAS*^G12C^ on ICI efficacy may be similar to the one of other *KRAS* mutations. Given this, the lack of *KRAS*^G12C^ assessment in the POPLAR/OAK cohort may not affect the main findings of our study. Second, the present study is strong at clinical analysis and relatively weak at exploring mechanism. Investigations into the biological mechanism of co-mutations are warranted in the future studies. Third, the validation cohorts are heterogenous in terms of clinical characteristics, ICIs given (especially anti-PD(L)1 monotherapy vs. combination with anti-CTLA-4), mutational status, treatment lines, and etc., which might affect the results. Of note, the training sets merely include patients treated with monotherapy. Despite that the inter-models performed well in the validation sets consisting of patients undergoing combination therapy, there may be room for improvement. Moreover, further validations by other cohorts are required. Fourth, the retrospective setting of our study may introduce biases, but this limitation can be greatly minimized by large sample size, multi-cohort methodology, and implementation of subgroup analysis and multivariable analysis, by which experimental features could be balanced (e.g., race, ICI regimen, treatment lines, and the platform/panel/used samples of next-generation sequencing [NGS] testing) and the possibility of confounding impact from these variables might be excluded to some extent.

To our knowledge, this is the first study delineating the landscape of interaction effects between co-occurring mutations on the ICI efficacy in NSCLC, which provides novel insights for basic research into the interactive impacts of co-occurring genomic alterations on tumor immunobiology and immune contexture. Furthermore, we developed and validated two cost-effective and quantitative prediction models involving key interaction terms to predict favorable benefit from ICI treatment in nonsquamous and squamous NSCLC more precisely, compared to the models without interaction terms. Our comparative analysis highlights the advantage and necessity of involving co-occurring genomic alterations for facilitating the refinement of prediction algorithms for cancer therapeutics.

## Methods

### Patients

Eight cohorts of 1745 NSCLC patients treated with ICI were analyzed^19–24,26,37^, from National Cancer Center (NCC), SYSUCC, DFCI, MSKCC, and the POPLAR/OAK trial, and TCGA database. ICI agent, setting, number of patients, treatment lines, outcome, PD-L1 antibody, NGS technique, and survival data are shown in Supplementary Table 1 and Supplementary Fig. 1. Of note, overlapped patients were identified among the separately published four MSKCC cohorts by the patient identifier (e.g., P-0003869). We merged these cohorts and then classified them into two subsets based on the used ICI agents (anti-PD-(L)1 monotherapy or combination therapy with anti-CTLA-4). The written consents were received from all the participated patients. Besides, the TCGA-LUAD and -LUSC datasets, and the results of immunogenomics analysis from Thorsson et al.^38^ were analyzed to explore the immune-related mechanism. Outcomes, analyzed mutations, and definition of TMB > median and PD-L1 positivity are in Supplemental Methods and Supplementary Table 2.

Our study is following the principles of the Declaration of Helsinki and approved by the Institution Review Board of Chinese PLA General Hospital (2015L01380). This report follows the Strengthening the Reporting of Observational Studies in Epidemiology (STROBE) reporting guideline.

### Statistical analysis

To assess the between-group difference, we performed (i) Fisher exact test and Chi-square test for categorical variables, (ii) Mann-Whitney tests for continuous variables, and (iii) Kaplan-Meier (KM) method, Log-rank method, and Cox regression (hazard ratio [HR] and 95% confidence interval [CI]) for survival variables. The variables with *p* value below 0.10 in the univariable analyses were included in the following multivariable analyses.

The area under the curve (AUC) of the ROC curve was calculated to estimate the discriminative performance. Multivariable Cox regression was performed to develop prediction models, and the scores were calculated for each patient using a formula derived from the mutation status (1 or 0) weighted by their regression coefficient: $score = {\sum} {(mutationstatus \times coefficient)}$. The coefficients of the models for nonsquamous and squamous NSCLC are displayed in Supplementary Table 8 and Supplementary Table 10, respectively. The nomograms are displayed in Supplementary Fig. 2 and Supplementary Fig. 6, respectively. The potential optimal cut-off was determined by the ROC curve of objective response in the training set. Calibration curves were drawn via bootstrap resampling 1000 times, and *D*_*xy*_ was calculated by Somers’ rank correlation between predicted log relative hazard and PFS. All statistical analyses mentioned above were performed using IBM SPSS Statistics 22 or R 3.4.2. The nominal level of significance was set as 5%, and all 95% CIs were 2-sided.

### Reporting summary

Further information on research design is available in the Nature Research Reporting Summary linked to this article.

## Supplementary information

Supplementary Information

Reporting Summary

## Data Availability

The authors declare that relevant data supporting the findings of this study are available within the paper and its Supplementary files. The source of the data (hyperlinks and DOIs) of all the included datasets are shown in Supplementary Table 1. The data of the NCC and SYSUCC cohorts are obtained through sending requests to the corresponding authors. Due to ethical and privacy concerns, we are sorry that we are unable to publish their full data in our study. Readers may contact the corresponding authors for the access of individual patient-level data for non-commercial purposes.

## Code availability

We used IBM SPSS Statistics 25 and R version 4.1.0 for data analysis. The packages we performed in R include “ggplot2”, “ggpubr”, “magrittr”, “survival”, “survminer”, “reshape2”, “forcats”, “rms”, “foreign”, “caret”, “PresenceAbsence”, “riskRegression”, and “prodlim”.
