# Supplementary material for: Co-occurring genomic alterations and immunotherapy efficacy in NSCLC
Source: NPJ Precis Oncol. 2022 Jan 18;6:4. doi: 10.1038/s41698-021-00243-7 (PMC8766442; doi:10.1038/s41698-021-00243-7)
Supplement: Supplementary file 1 — Supplementary Information [file 41698_2021_243_MOESM1_ESM.pdf]

Supplemental Materials

Supplement to: F. Zhang, L. Zhao, T. Li, Y. Xu, et al.

Co-occurring genomic alterations impact immunotherapy efficacy in NSCLC

TABLE OF CONTENTS

Supplemental Methods 2

Outcomes 2

Mutational analysis 2

Define the cut-off of the quantity of co-occurring mutations 2

Definition of TMB>median and PD-L1 positivity in subgroup analysis and multivariable analysis 3

Cell-type identification by estimating relative subsets of RNA transcripts (CIBERSORT) 3

Analysis of predicted neoantigen load 3

Figure legends of Supplemental Figures 4

Supplemental Tables 17

Supplementary Table 1. Clinical cohorts analyzed in this study. 17

Supplementary Table 2. Members of the analyzed signaling pathways. 18

Supplementary Table 3. The single mutations associated with PFS on anti-PD-(L)1 monotherapy in non-squamous NSCLCs. 19

Supplementary Table 4. The interaction effects of co-mutations associated with PFS on anti-PD-(L)1 monotherapy in non-squamous NSCLCs. 20

Supplementary Table 5. The single mutations associated with PFS on anti-PD-(L)1 monotherapy in squamous NSCLCs. 21

Supplementary Table 6. The interaction effects of co-occurring mutations associated with PFS on anti-PD-(L)1 monotherapy in non-squamous NSCLCs. 22

Supplementary Table 7. Univariable-significant single mutational events and significant interactions based on the PFS data of the non-squamous NSCLCs in the training sets. 23

Supplementary Table 8. Three prediction models based on the PFS on anti-PD-(L)1 monotherapy of the non-squamous NSCLCs in the training sets. 24

Supplementary Table 9. Univariable-significant single mutational events and significant interactions based on the PFS data of the squamous NSCLCs in the training sets. 25

Supplementary Table 10. Three prediction models based on the PFS on anti-PD-(L)1 monotherapy of the squamous NSCLCs in the training sets. 26

References 27

Supplemental Methods

Outcomes

Objective response rate (ORR) was defined as the percentage of patients with confirmed complete response (CR) or partial response (PR) according to Response Evaluation Criteria in Solid Tumors, version 1.1 (RECIST v1.1) by thoracic radiologists or investigators. PFS was defined as the time from the start of treatment until disease progression (RECIST v1.1) or death from any cause. Overall survival (OS) was defined as the time from the start of treatment to death or the date of the last follow-up.

Mutational analysis

Mutated genes included in our analysis were restricted to non-silent mutations consisting of nonsense mutation, missense mutation, frameshift mutation, inframe mutation, splice site mutation, translation start site mutation, and nonstop mutation. Truncating mutations of oncogene were excluded because most of these are passenger mutations with limited cancer-promoting function.

The signaling pathways and their members analyzed in the present study are shown in **Supplemental Table S2**. This idea of pathway-level genomic alterations was derived from multi-omics studies[1-3]. Moreover, a study using similar concept was conducted to investigate the genomic correlates of PD-L1 expression in patients with lung adenocarcinomas [4]. The definition of pathways in the present study drew upon these previous works. In addition, we selected the genes that were detected in all the included cohorts.

Define the cut-off of the quantity of co-occurring mutations

To reduce the sampling error of the data displayed in **Fig. 1**, we needed to set a cut-off value of the frequency of co-occurring mutations. The training cohort of non-squamous NSCLC (n=592) consists of two meta-datasets (1: MSKCC/DFCI/SYSUCC [n=288], 2: POPLAR/OAK [304]). We calculated the interaction effect of each co-mutated pair in each meta-dataset, and use the formula below to assess the difference between the interaction effects of each pair in the two meta-datasets.

$$Difference\ value = Absolute\ value\ of\ log_2(HR_{training\ set\ 1}/HR_{training\ set\ 2})$$

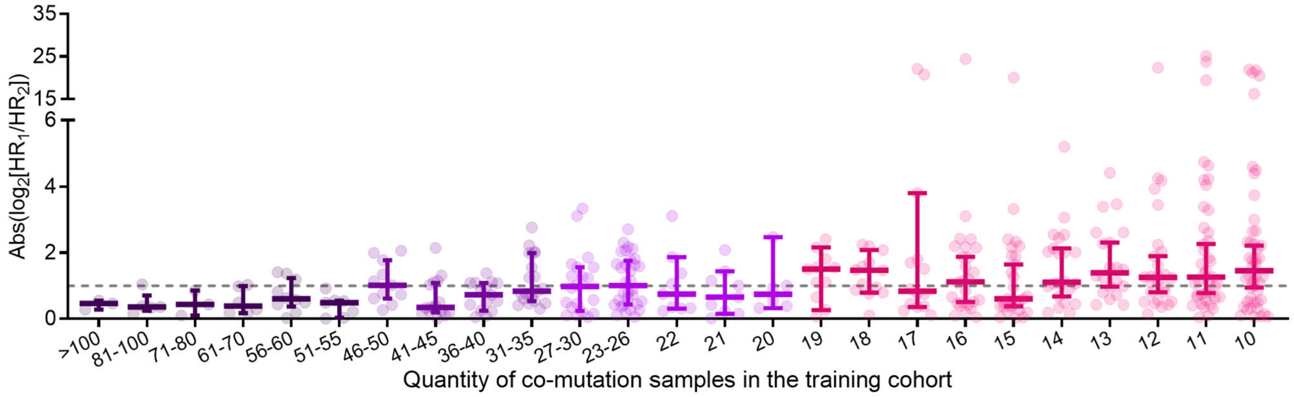

The medians of the difference value were mostly below 1 in the groups with ≥20 quantities of co-mutation samples, and the medians of the groups “quantity=19” and “quantity=18” increased to 1.51 and 1.47,

respectively. Moreover, several difference values over 15 were observed in the groups with ≤17 quantities of co-mutation samples. Taken together, these results indicate that the co-mutations existing in ≥20 patients in the training cohort exhibited more robustly in predicting ICI efficacy, and therefore we set the cut-off of the quantity of co-mutation as 20.

Definition of TMB>median and PD-L1 positivity in subgroup analysis and multivariable analysis

As for TMB, the panels and the tested samples (tissue/blood) are not the same in different cohorts (**Supplemental Table S1**), but the distribution and the order of included patients may be largely similar owing to the high correlations between tTMB and bTMB and between different panels[5-7]. The similarity of distribution was further exemplified by the scatter plots in **Fig. 3J-L**. Given this, we used the median value in each cohort to classify the patients into the TMB<median and the TMB≥median subgroups, and then separately combined all the TMB<median subgroups and all the TMB≥median subgroups for further analysis.

Despite that the PD-L1 antibodies used for immunohistochemistry in the included cohorts are not identical (**Supplemental Table S1**), a combined analysis of PD-L1 positivity was implemented due to the concordance among these antibodies[8,9].

Cell-type identification by estimating relative subsets of RNA transcripts (CIBERSORT)

CIBERSORT, an online method (https://cibersort.stanford.edu/index.php) for characterizing cell composition of complex tissues from their gene expression profiles[10], was applied to the enumeration of hematopoietic subsets in mRNA mixtures from TCGA database. CIBERSORT outperformed other methods with respect to noise, unknown mixture content, and closely related cell types[10].

Analysis of predicted neoantigen load

The data of predicted neoantigen load were retrieved from the MSKCC-34 and MSKCC-75 cohorts[11,12]. We performed Spearman correlation analysis to assess the association between the inter-score and neoantigen load.

Supplemental Figures

Supplementary Fig. 1. PFS data of the included patients from different sources.

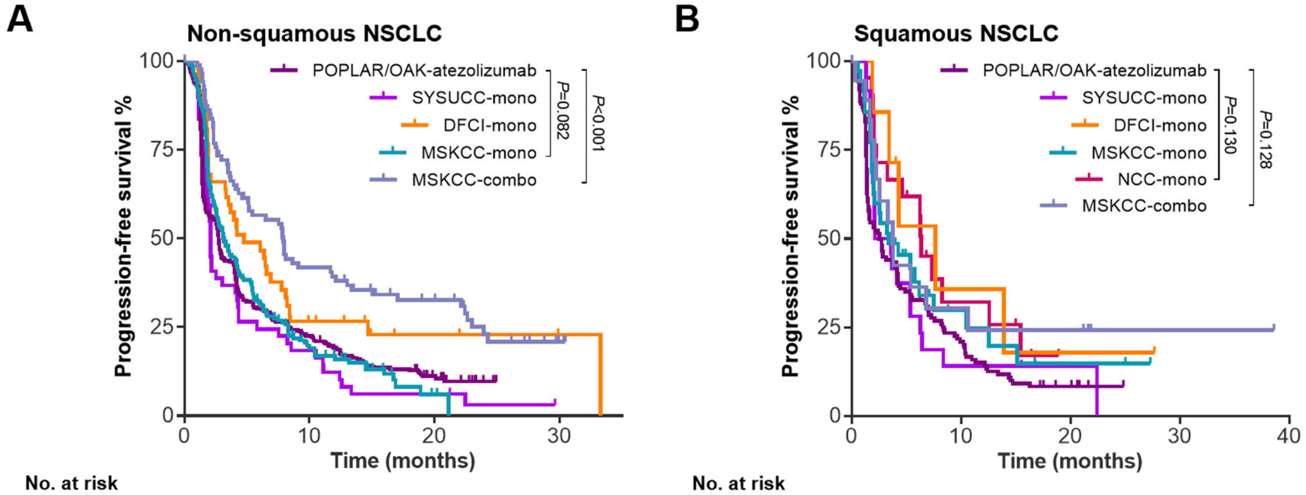

No. at risk (A)

POPLAR/OAK	304	68	18	0
SYSUCC	49	9	3	0
DFCI	47	10	3	1
MSKCC-mono	192	27	2	0
MSKCC-combo	89	34	20	3

No. at risk (B)

POPLAR/OAK	125	25	7	0
SYSUCC	24	3	1	0
DFCI	7	2	1	0
MSKCC-mono	35	6	2	0
NCC-mono	21	5	0	0
MSKCC-combo	19	5	4	1

A-B. KM curves illustrating the PFS data from different sources of non-squamous and squamous NSCLC. Abbreviations: combo=combination therapy with anti-cytotoxic T lymphocyte antigen-4, DFCI=Dana Farber Cancer Institute, KM=Kaplan-Meier, mono=anti-programmed death-(ligand) 1 monotherapy, MSKCC=Memorial Sloan-Kettering Cancer Center, NCC=National Cancer Center, NSCLC=non-small cell lung cancer, PFS=progression-free survival, SYSUCC=Sun Yat-Sen University Cancer Center.

Supplementary Fig. 2. Nomograms of the three models predicting the PFS on ICI therapy in the patients with non-squamous NSCLC.

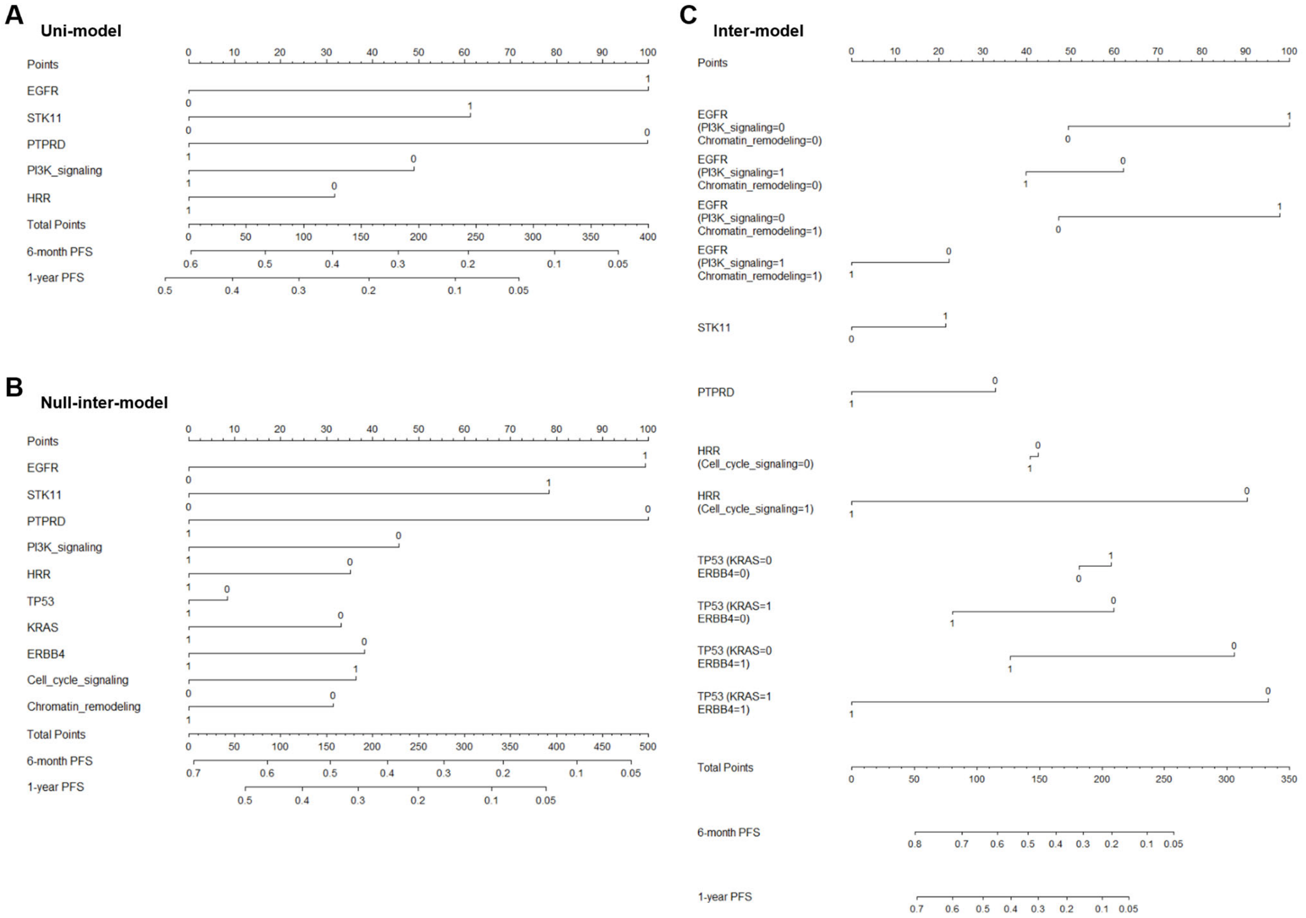

A-**C**. The nomograms of the uni-model (**A**), the null-inter-model (**B**), and the inter-model (**C**). Abbreviations: ICI=immune checkpoint inhibitor, NSCLC=non-small cell lung cancer.

Supplementary Fig. 3. Performances of the three models in predicting objective response to ICI therapy in non-squamous NSCLC.

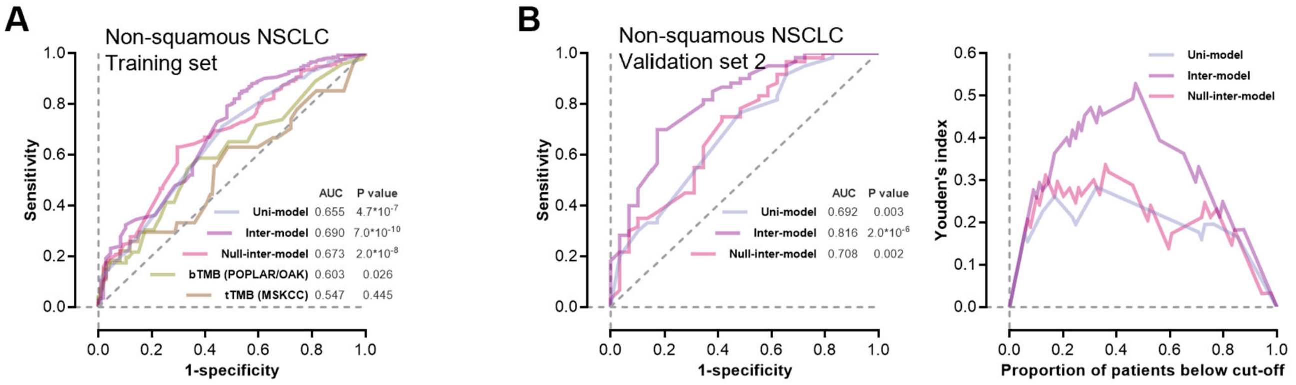

A. The ROC curves of response and the three scores, blood TMB, and tissue TMB in the training sets-1/2 of non-squamous NSCLC. **B**. The ROC curves of response and the three scores in the validation set-2 of non-squamous NSCLC (left) and the Youden's indices based on these ROC curves (right). Abbreviations: ICI=immune checkpoint inhibitor, NSCLC=non-small cell lung cancer, ROC=receiver operating characteristic, TMB=tumor mutational burden.

Supplementary Fig. 4. Association between scores and prognosis in the POPLAR/OAK-docetaxel cohort and the TCGA-LUAD cohort.

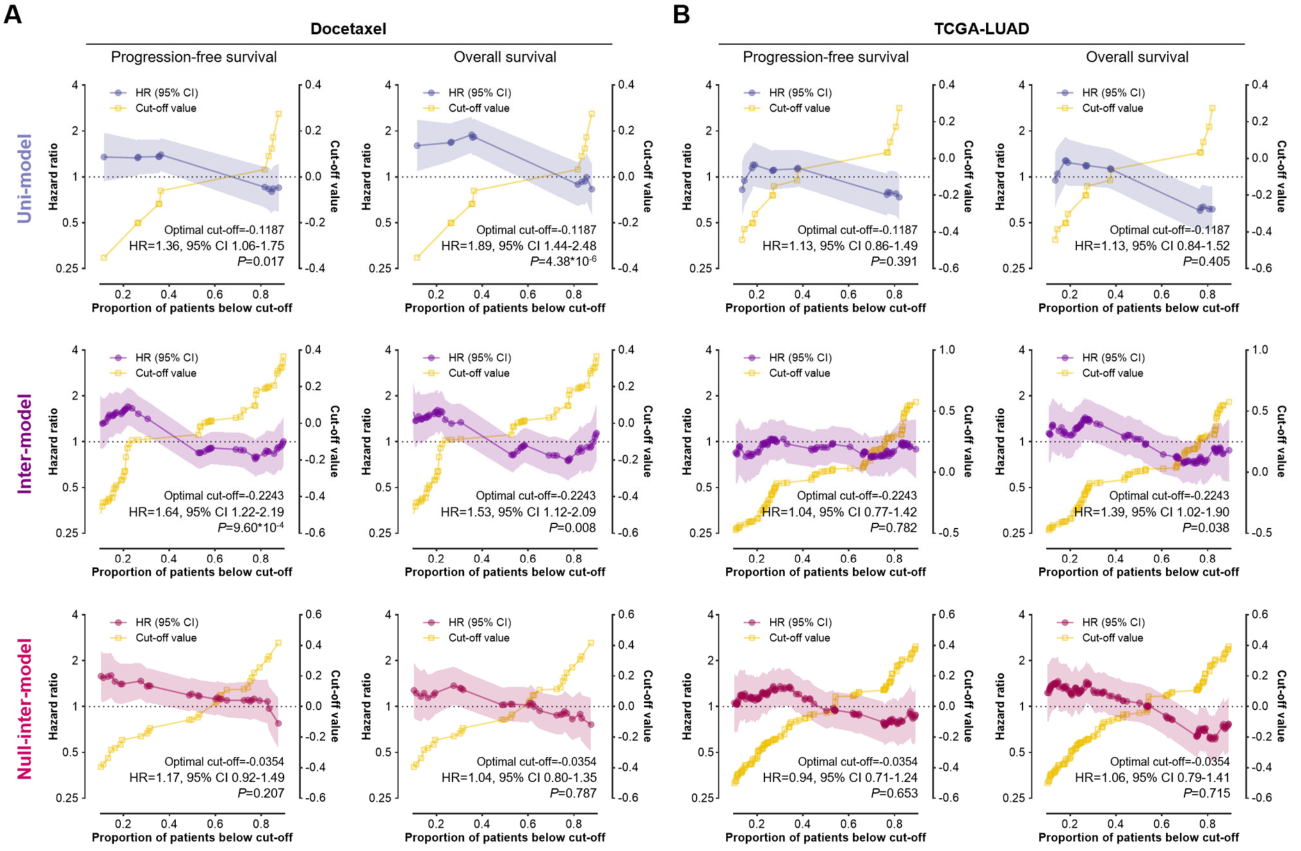

A-B. Associations between scores and survival outcomes with docetaxel (**A**) and prognosis in the TCGA cohort (**B**). Abbreviations: LUAD=lung adenocarcinoma, NSCLC=non-small cell lung cancer, TCGA=The Cancer Genomic Atlas.

Supplementary Fig. 5. Workflow of developing and validating three models in squamous NSCLC.

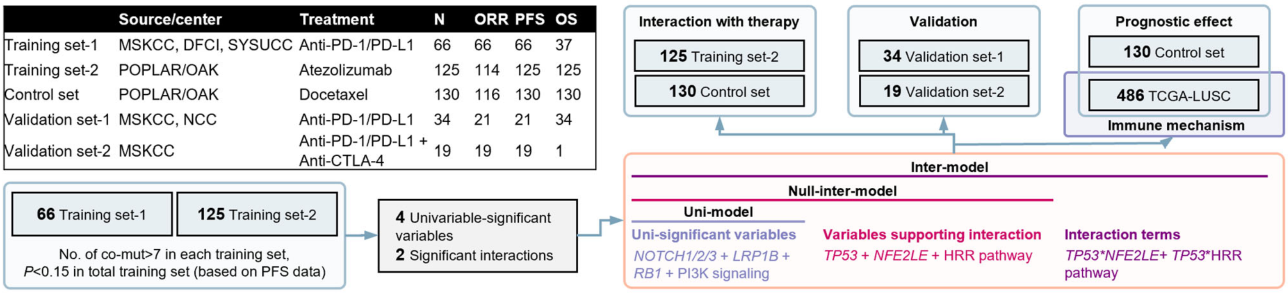

	Source/center	Treatment	N	ORR	PFS	OS
Training set-1	MSKCC, DFCI, SYSUCC	Anti-PD-1/PD-L1	66	66	66	37
Training set-2	POPLAR/OAK	Atezolizumab	125	114	125	125
Control set	POPLAR/OAK	Docetaxel	130	116	130	130
Validation set-1	MSKCC, NCC	Anti-PD-1/PD-L1	34	21	21	34
Validation set-2	MSKCC	Anti-PD-1/PD-L1 + Anti-CTLA-4	19	19	19	1

Abbreviations: DFCI=Dana Farber Cancer Institute, MSKCC=Memorial Sloan-Kettering Cancer Center, NCC=National Cancer Center, NSCLC=non-small cell lung cancer, OS=overall survival, PD-1=programmed death-1, PD-L1=programmed death-ligand 1, PFS=progression-free survival, SYSUCC=Sun Yat-Sen University Cancer Center.

Supplementary Fig. 6. Nomograms of the three models predicting the PFS on ICI therapy in the patients with squamous NSCLC.

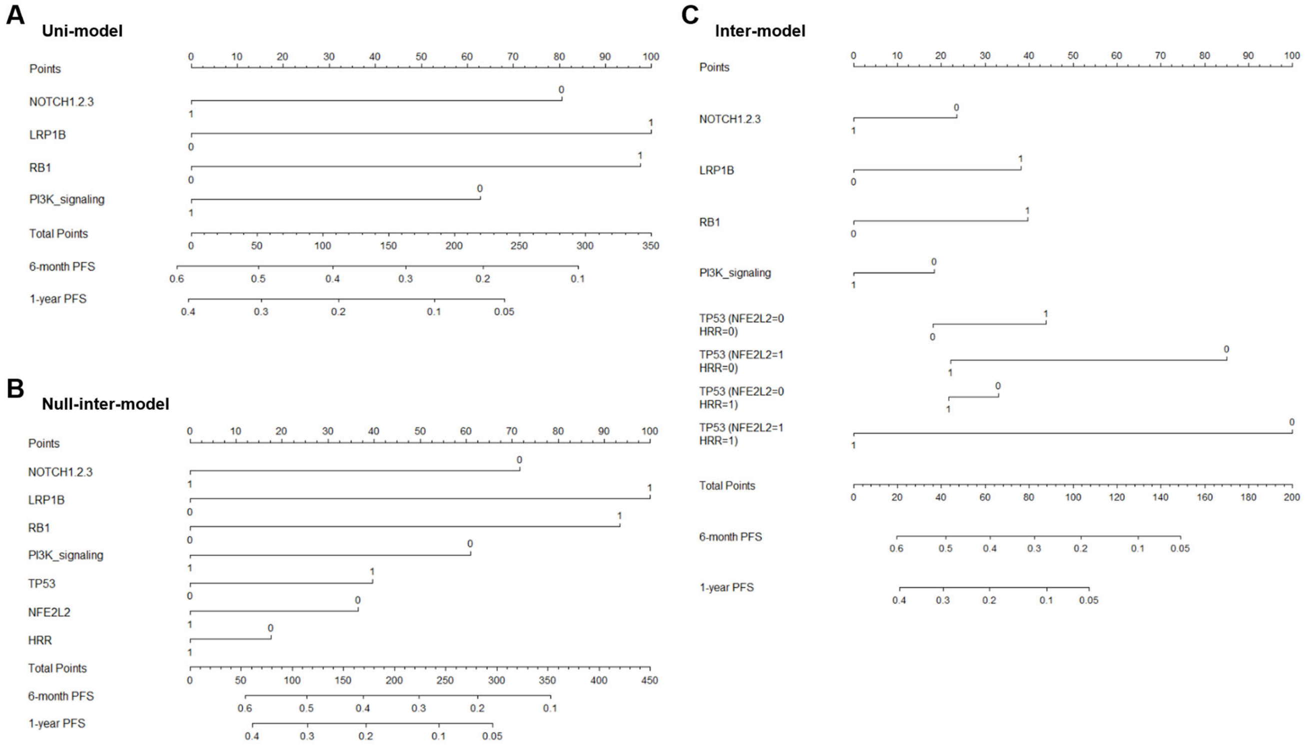

A-C. The nomograms of the uni-model (**A**), the null-inter-model (**B**), and the inter-model (**C**). Abbreviations: ICI=immune checkpoint inhibitor, NSCLC=non-small cell lung cancer.

Supplementary Fig. 7. Performances of the three models in predicting objective response to ICI therapy in squamous NSCLC.

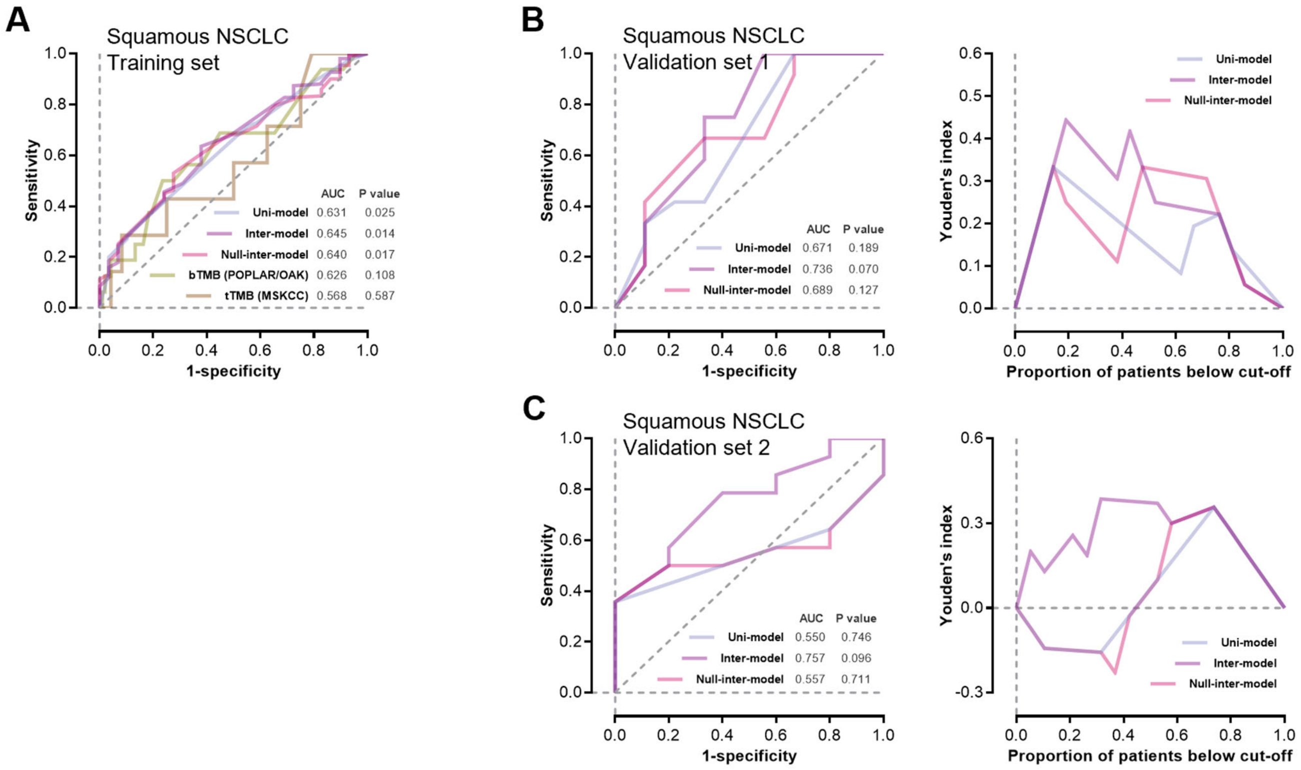

A. The ROC curves of response and the three scores, blood TMB, and tissue TMB in the training sets-1/2 of squamous NSCLC. **B**. The ROC curves of response and the three scores in the validation set-1 of non-squamous NSCLC (left) and the Youden's indices based on these ROC curves (right). **C**. The ROC curves of response and the three scores in the validation set-2 of non-squamous NSCLC (left) and the Youden's indices based on these ROC curves (right). Abbreviations: ICI=immune checkpoint inhibitor, NSCLC=non-small cell lung cancer, ROC=receiver operating characteristic, TMB=tumor mutational burden.

Supplementary Fig. 8. Performances of the three-models in predicting PFS/OS on immunotherapy and PFS/OS benefit from atezolizumab over docetaxel in the training sets of squamous NSCLC.

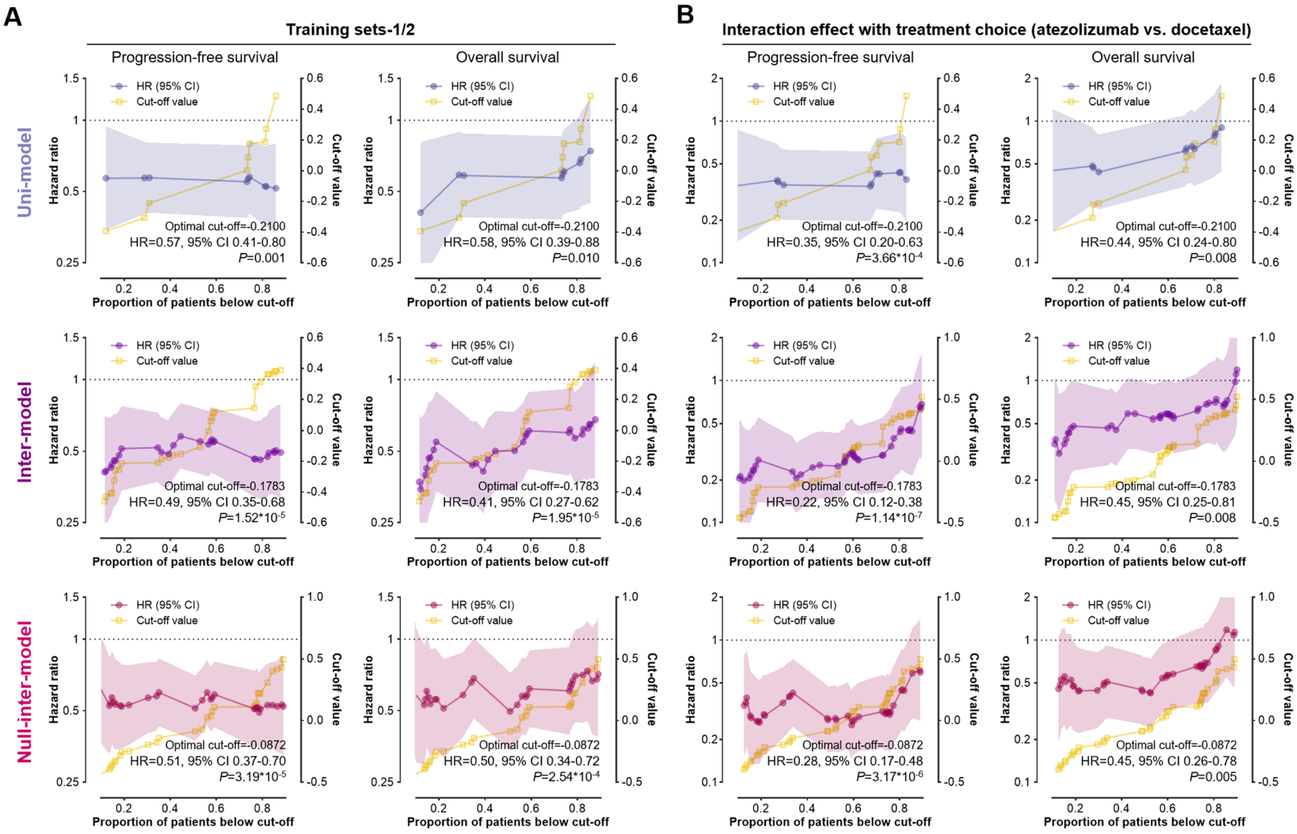

A. Performances of the three-models on discriminating the PFS and OS on anti-PD-(L)1 monotherapy in the training sets-1/2. **B**. Performances of the three-models on predicting the PFS and OS benefit from atezolizumab over docetaxel in the POPLAR/OAK cohort. Abbreviations: NSCLC=non-small cell lung cancer, OS=overall survival, PD-1=programmed death-1, PD-L1=programmed death-ligand 1, PFS=progression-free survival.

Supplementary Fig. 9. Performances of the three models in predicting PFS/OS on ICI treatment in the validation sets of squamous NSCLC.

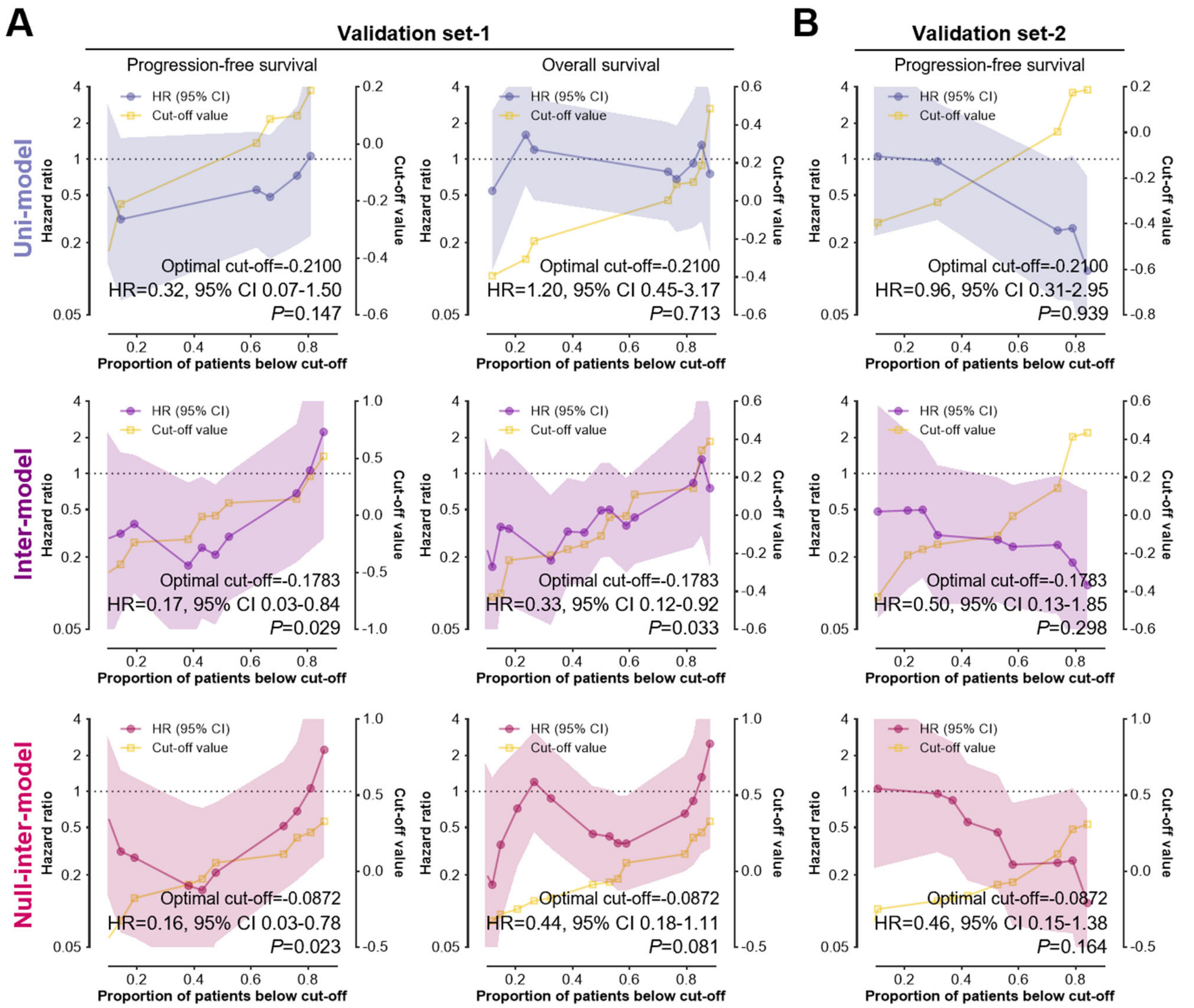

A-B. Performances of the three-models on discriminating the OS on anti-PD-(L)1 monotherapy in the validation set-1 (**A**) and the PFS on combination therapy with anti-CTLA-4 in the validation set-2 (**B**). Abbreviations: OS=overall survival, PD-1=programmed death-1, PD-L1=programmed death-ligand 1, PFS=progression-free survival.

Supplementary Fig. 10. Association between scores and prognosis in the POPLAR/OAK-docetaxel cohort of squamous NSCLC and the TCGA-LUSC cohort.

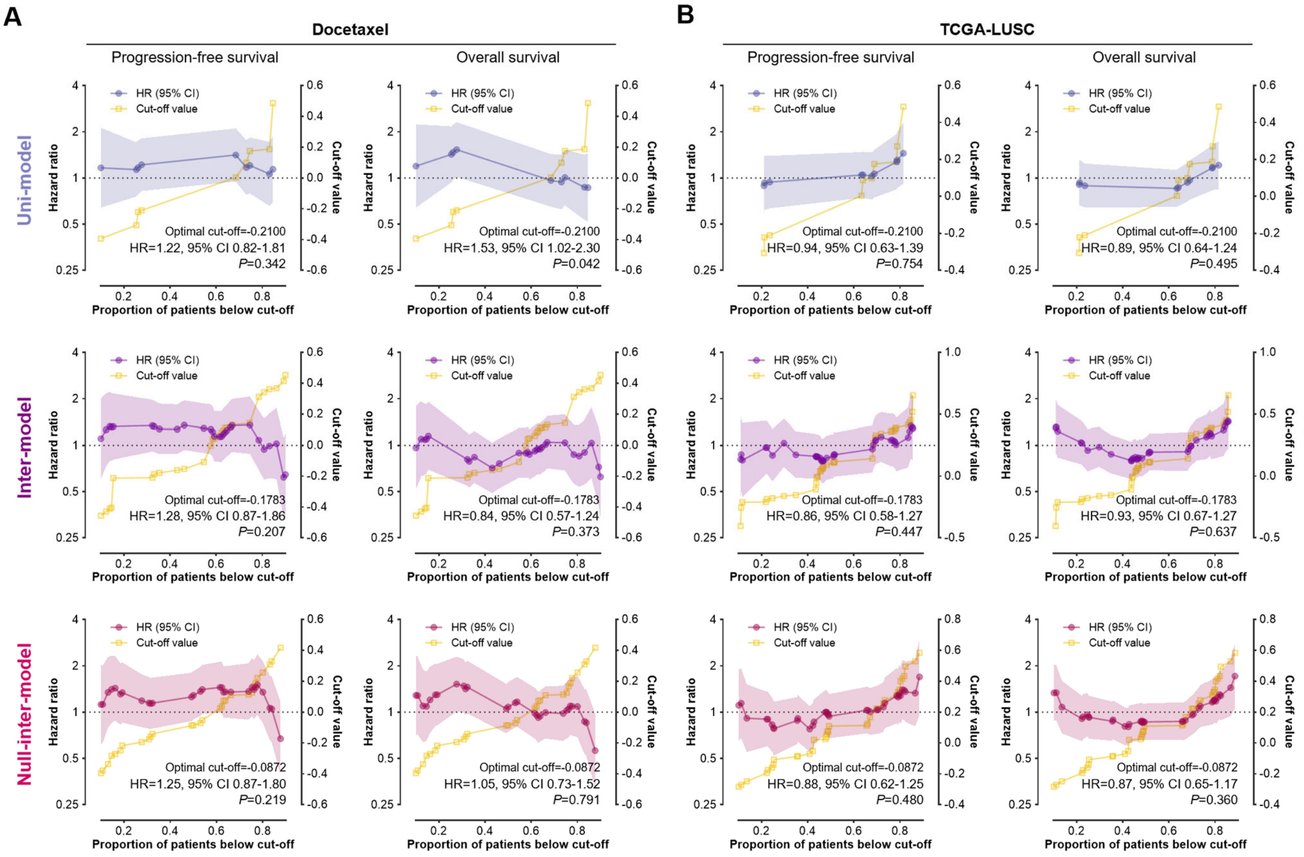

A-B. Associations between scores and survival outcomes with docetaxel (**A**) and prognosis in the TCGA cohort (**B**). Abbreviations: LUSC=lung squamous cell carcinoma, NSCLC=non-small cell lung cancer, TCGA=The Cancer Genomic Atlas.

Supplementary Fig. 11. Immune correlates of the inter-score in the TCGA-LUSC cohort.

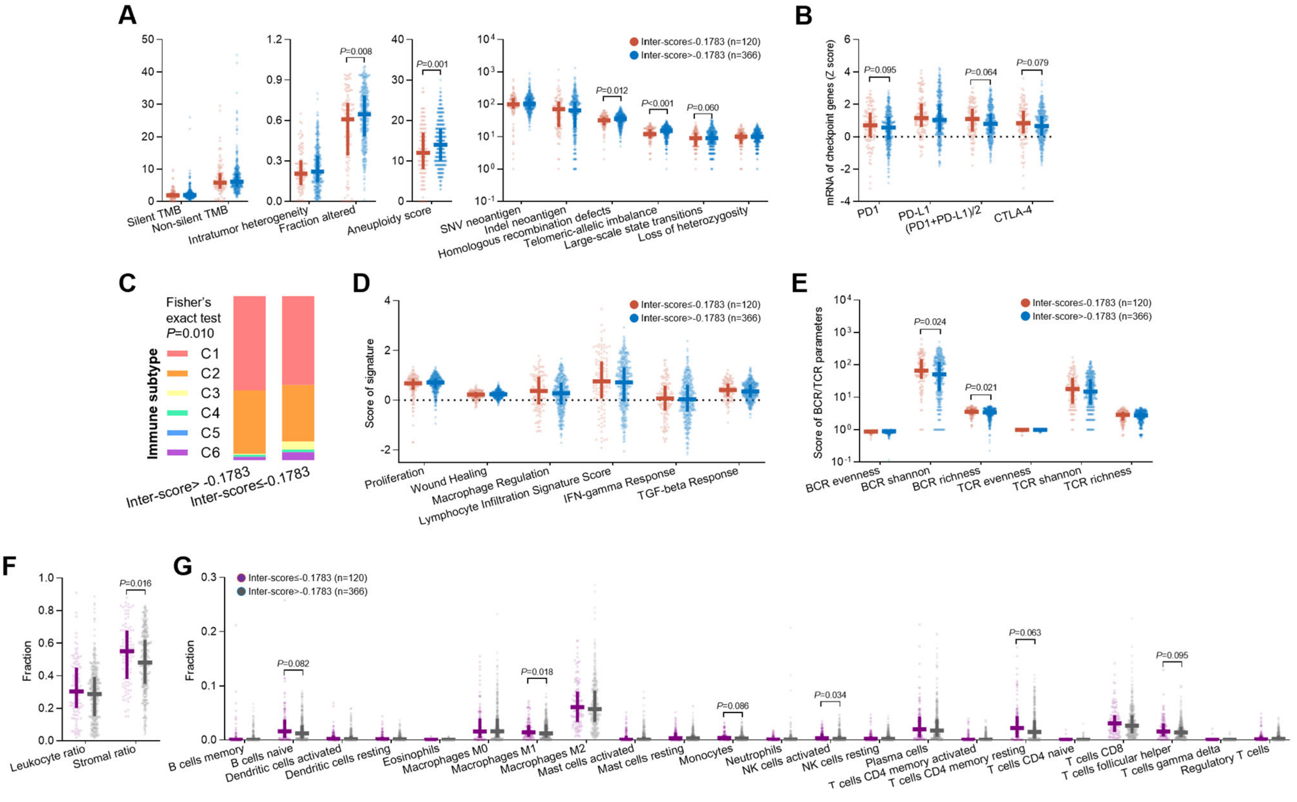

A-G. Associations of the inter-score with TMB, intratumor heterogeneity, fraction altered, aneuploidy score, neoantigen load, homologous recombination defects (**A**), mRNA of immune checkpoint genes (**B**), immune subtype (**C**), signatures supporting immune subtyping (**D**), and BCR/TCR parameters (**E**), leukocyte/stromal ratio (**F**), and tumor-infiltrating immune cells (**G**). Abbreviations: BCR=B cell receptor, LUSC=lung squamous cell carcinoma, SNV=single nucleotide variation, TCGA=The Cancer Genomic Atlas, TCR=T cell receptor, TMB=tumor mutational burden.

Supplementary Fig. 12. Association of *KEAP1* mutation and *KRAS*-G12C mutation with immunotherapy efficacy in non-squamous NSCLC.

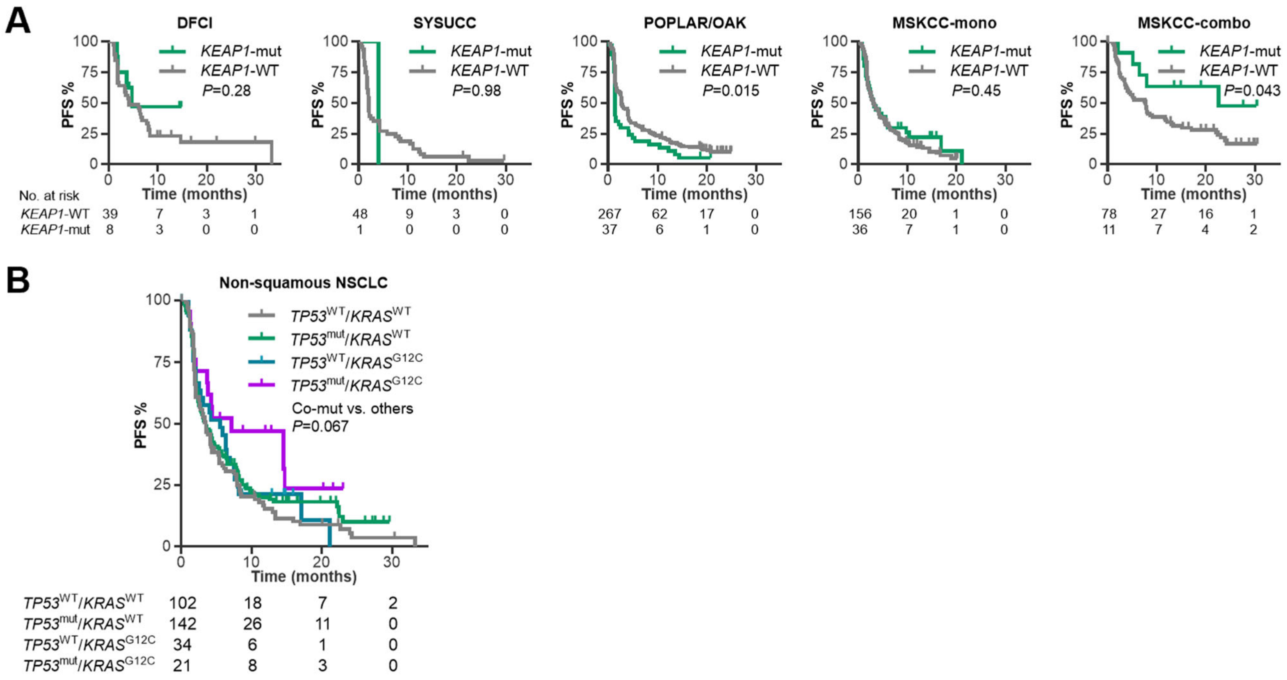

A. Association between *KEAP1* mutation and immunotherapy efficacy in non-squamous NSCLC. **B**. Association between *TP53*/*KRAS* co-mutation and immunotherapy efficacy in non-squamous NSCLC. Abbreviations: NSCLC=non-small cell lung cancer, PFS=progression-free survival.

Supplementary Fig. 13. Association of the inter-score with tissue TMB≥10 and PD-L1≥50% in predicting the PFS on ICI treatment in the patients with NSCLC.

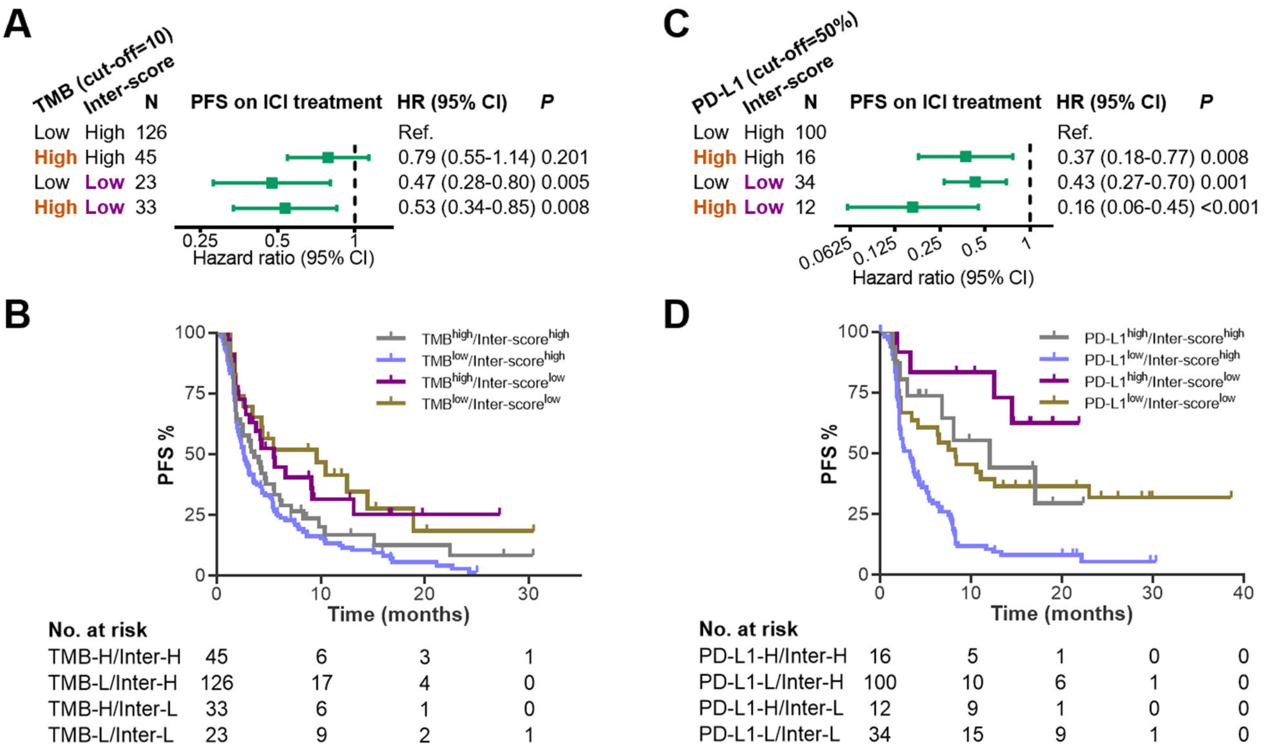

A-B. Predictive effectiveness of the inter-scores in the NSCLC patients with TMB≥10 (**A**) or PD-L1≥50% (**B**). Abbreviations: ICI=immune checkpoint inhibitor, NSCLC=non-small cell lung cancer, PD-L1=programmed death-ligand 1, PFS=progression-free survival, TMB=tumor mutational burden.

Supplemental Tables

Supplementary Table 1. Clinical cohorts analyzed in this study.

Cohort	NCC	SYSUCC	DFCI	POPLAR/OAK	MSKCC-34	MSKCC-240	MSKCC-75	MSKCC-350
**Journal**	*JAMA Oncol* [5]	*CCR* [13]	*Nat Genet* [14]	*Nat Med* [6]	*Science* [11]	*JCO* [7]	*Cancer Cell* [12]	*Nat Genet* [15]
**Year**	2019	2019	2018	2018	2015	2018	2018	2019
**Regimen**	Anti-PD-1/PD-L1	Anti-PD-1/PD-L1	Anti-PD-1/PD-L1	Atezolizumab vs. docetaxel (RCT, no crossover)	Pembrolizumab	Anti-PD-1/PD-L1, or combined with anti-CTLA-4	Nivolumab + Ipilimumab	Anti-CTLA4, anti-PD-1/PD-L1 or combination
**Setting**	Real-world	Real-world	Not mentioned	Clinical trial	Clinical trial	Clinical trial and real-world	Clinical trial	Clinical trial and real-world
**Cancer type**	NSCLC	NSCLC	Pan-cancer	NSCLC	NSCLC	NSCLC	NSCLC	Pan-cancer
**No. of NSCLC pts**	64	73	56	853	34	240	75	350
**Treatment lines**	First to subsequent	First to subsequent	Not mentioned	Second/third	Not mentioned	First to subsequent	Not mentioned	Not mentioned
**Outcome**	ORR, PFS, OS	ORR, PFS	ORR, PFS, OS	ORR, PFS, OS	ORR, PFS	ORR, PFS	ORR, PFS	OS
**PD-L1 IHC testing**	Ventana SP263	Dako 22C3	/	Ventana SP142 (in OAK trial)	Dako 22C3	/	Dako 28-8	/
**NGS testing**	Blood sample	Tissue sample	Tissue sample	Blood sample	Tissue sample	Tissue sample	Tissue sample	Tissue sample
	SNV	SNV	SNV	SNV	SNV	SNV, CNV, fusion	SNV	SNV, fusion
	3D Medicines panel: 150-gene	Whole-exome sequencing	Whole-exome sequencing	Foundation One panel: 315-gene, 1.1 Mb	Whole-exome sequencing	MSKCC panel: 341-gene, 56 pts; 410-gene, 164 pts; 468-gene, 20 pts	Whole-exome sequencing	MSKCC panel: 341-gene, 56 pts; 410-gene, 239 pts; 468-gene, 55 pts
**Source of data**	●Sending request to the corresponding authors	●Sending request to the corresponding authors	●Supplemental Materials ●Newly sequenced samples are available at dbGaP under accession number phs001565.v1.p1.	●Supplemental Materials ●https://clinicalstudydatarequest.com/).	●http://science.sciencemag.org/content/suppl/2015/03/11/science.aaa1348.DC1 ●http://www.cbioportal.org/study?id=tmb_mskcc_2018.	●Supplemental Materials ●http://www.cbioportal.org/study?id=tmb_mskcc_2018.	●Supplemental Materials ●https://www.ebi.ac.uk/eva/?evastudy=PRJEB24995 ●http://www.cbioportal.org/study?id=tmb_mskcc_2018.	●Supplemental Materials ●http://www.cbioportal.org/study?id=tmb_mskcc_2018.

Abbreviations: CNV=copy number variation, CTLA-4=cytotoxic T lymphocyte-associated antigen 4, DFCI=Dana Farber Cancer Institute, MSKCC=Memorial Sloan-Kettering Cancer Center, IHC=immunohistochemistry, NCC=National Cancer Center, NGS=next-generation sequencing, NSCLC=non-small cell lung cancer, ORR=overall response rate, OS=overall survival, PD-1=programmed death 1, PD-L1=programmed death-ligand 1, PFS=progression-free survival, SNV=single nucleotide variation, SYSUCC=Sun Yat-Sen University Cancer Center.

Supplementary Table 2. Members of the analyzed signaling pathways.

Pathway	Gene list
RAS pathway	*KRAS NRAS BRAF*
NOTCH pathway	*NOTCH1 NOTCH2 NOTCH3 SPEN EP300 FBXW7 KDM5A*
WNT pathway	*LRP1B APC CTNNB1*
Cell cycle pathway	*RB1 CDKN2A JAK1 JAK2*
PI3K pathway	*PIK3CA PTEN TSC1 TSC2 AKT1 AKT2 AKT3 MTOR RICTOR RPTOR*
RTK pathway	*ERBB2 ERBB3 ERBB4 PDGFRA PDGFRB KDR FGFR1 FGFR2 FGFR3 FGFR4*
TGF-beta pathway	*SMAD2 SMAD3 SMAD4 INHBA TGFBR2*
HRR pathway	*ATM BRCA1 BRCA2 PALB2 CDK12 ATR MUTYH*
SWI/SNF pathway	*ARID1A ARID1B ARID2 PBRM1 SMARCA4*
Chromatin remodeling pathway	*SETD2 DOT1L EZH2*
Hippo pathway	*FAT1 FAT4 NF1 NF2*
Hedgehog pathway	*GLI1 SMO*
Other involved genes	*EGFR ALK TP53 STK11 KEAP1 NFE2L2 RBM10 PTPRD*

Abbreviations: HRR=homologous recombination repair, RTK=receptor tyrosine kinase, SWI/SNF=switch-sucrose nonfermentable, TGF=transforming growth factor.

Supplementary Table 3. The single mutations associated with PFS on anti-PD-(L)1 monotherapy in non-squamous NSCLCs.

	No. of mutated samples	HR (95% CI)	P value
EGFR	91	1.53 (1.19-1.98)	0.001
STK11	115	1.31 (1.03-1.67)	0.026
PTPRD	82	0.56 (0.42-0.75)	<0.001
NOTCH pathway	109	0.72 (0.56-0.92)	0.009
NOTCH1/2/3	70	0.72 (0.54-0.96)	0.025
LRP1B	116	0.77 (0.62-0.97)	0.025
PI3K pathway	145	0.77 (0.62-0.96)	0.022
RTKs	153	0.73 (0.59-0.90)	0.004
SMAD4	28	1.60 (1.01-2.53)	0.046
HRR pathway	163	0.77 (0.63-0.95)	0.016
ATM	64	0.72 (0.54-0.97)	0.029
ATR	28	0.62 (0.39-1.00)	0.049
ARID2	54	0.69 (0.49-0.97)	0.032
Hippo pathway	143	0.78 (0.62-0.97)	0.023
Hedgehog pathway	36	0.60 (0.37-0.96)	0.032

Abbreviations: CI=confidence interval, HR=hazard ratio, NSCLC=non-small cell lung cancer, PD-1=programmed death-1, PD-L1=programmed death-ligand 1, PFS=progression-free survival.

Supplementary Table 4. The interaction effects of co-mutations associated with PFS on anti-PD-(L)1 monotherapy in non-squamous NSCLCs.

Mutational event 1	Mutational event 2	Interaction			Mutational event 1			Mutational event 2		
		No. of co-mutation samples	HR (95% CI)	P value	No. of event 1	HR (95% CI)	P value	No. of event 2	HR (95% CI)	P value
EGFR	PI3K pathway	23	0.43 (0.23-0.80)	0.008	68	2.06 (1.54-2.76)	<0.001	122	0.84 (0.66-1.07)	0.156
TP53	*KRAS/HRAS*	82	0.58 (0.37-0.88)	0.012	272	1.03 (0.84-1.25)	0.781	101	1.10 (0.84-1.43)	0.493
TP53	*KRAS*	74	0.51 (0.32-0.80)	0.003	280	1.04 (0.86-1.27)	0.685	101	1.10 (0.84-1.43)	0.493
TP53	*ERBB4*	30	0.36 (0.17-0.79)	0.01	324	0.98 (0.82-1.17)	0.795	12	1.58 (0.86-2.90)	0.137
TP53	*ARID1A*	21	0.26 (0.11-0.64)	0.003	333	0.97 (0.81-1.16)	0.767	19	1.46 (0.89-2.39)	0.130
TP53	*NF1*	45	0.50 (0.27-0.92)	0.026	309	0.99 (0.83-1.19)	0.924	24	1.14 (0.73-1.78)	0.562
PTPRD	*KRAS/HRAS*	29	2.38 (1.28-4.41)	0.006	53	0.43 (0.30-0.63)	<0.001	154	0.79 (0.63-0.99)	0.038
PTPRD	*KRAS*	29	2.47 (1.33-4.61)	0.004	53	0.43 (0.30-0.62)	<0.001	146	0.76 (0.60-0.95)	0.018
PTPRD	WNT pathway	25	0.48 (0.26-0.91)	0.025	57	0.74 (0.52-1.06)	0.103	127	1.05 (0.85-1.31)	0.638
RAS	*NOTCH1/2/3*	26	0.53 (0.28-1.00)	0.049	157	0.98 (0.79-1.23)	0.881	44	0.91 (0.65-1.27)	0.569
KRAS	*NOTCH1/2/3*	25	0.50 (0.26-0.96)	0.036	150	0.96 (0.77-1.20)	0.725	45	0.92 (0.66-1.28)	0.625
NOTCH pathway	Cell cycle pathway	21	0.39 (0.19-0.82)	0.013	88	0.82 (0.63-1.07)	0.143	58	1.41 (1.03-1.92)	0.030
WNT pathway	SWI/SNF	63	0.61 (0.39-0.95)	0.029	89	1.06 (0.82-1.35)	0.666	124	1.11 (0.86-1.43)	0.412
LRP1B	SWI/SNF	50	0.57 (0.35-0.93)	0.024	66	0.95 (0.72-1.25)	0.726	137	1.12 (0.88-1.42)	0.345
Cell cycle pathway	HRR	29	0.26 (0.14-0.49)	<0.001	50	1.97 (1.41-2.75)	<0.001	134	0.91 (0.73-1.14)	0.429

Abbreviations: CI=confidence interval, HR=hazard ratio, HRR=homologous recombination repair, NSCLC=non-small cell lung cancer, PD-1=programmed death-1, PD-L1=programmed death-ligand 1, PFS=progression-free survival, RTK=receptor tyrosine kinase.

Supplementary Table 5. The single mutations associated with PFS on anti-PD-(L)1 monotherapy in squamous NSCLCs.

	No. of mutated samples	HR (95% CI)	P value
NOTCH1/2/3	31	0.65 (0.41-1.00)	0.053
LRP1B	45	1.56 (1.09-2.24)	0.016
RB1	14	1.77 (1.00-3.15)	0.051
PI3K pathway	63	0.75 (0.53-1.05)	0.092

Abbreviations: CI=confidence interval, HR=hazard ratio, NSCLC=non-small cell lung cancer, PD-1=programmed death-1, PD-L1=programmed death-ligand 1, PFS=progression-free survival.

Supplementary Table 6. The interaction effects of co-occurring mutations associated with PFS on anti-PD-(L)1 monotherapy in non-squamous NSCLCs.

Mutational event 1	Mutational event 2	Interaction			Mutational event 1			Mutational event 2		
		No. of co-mutation samples	HR (95% CI)	P value	No. of event 1	HR (95% CI)	P value	No. of event 2	HR (95% CI)	P value
TP53	*NFE2L2*	28	0.21 (0.06-0.74)	0.016	107	1.37 (0.97-1.92)	0.071	3	3.71 (1.15-12.00)	0.029
TP53	HRR pathway	30	0.55 (0.25-1.19)	0.129	105	1.43 (0.99-2.06)	0.055	15	1.31 (0.70-2.43)	0.396
PI3K pathway	Hippo pathway	25	0.57 (0.27-1.18)	0.130	38	0.91 (0.59-1.39)	0.664	28	1.28 (0.83-1.98)	0.266

Abbreviations: CI=confidence interval, HR=hazard ratio, NSCLC=non-small cell lung cancer, PD-1=programmed death-1, PD-L1=programmed death-ligand 1, PFS=progression-free survival.

Supplementary Table 7. Univariable-significant single mutational events and significant interactions based on the PFS data of the non-squamous NSCLCs in the training sets.

	Mutational event 1	
Mutational event 1	HR (95% CI)	P value
EGFR	1.53 (1.19-1.98)	0.001
STK11	1.31 (1.03-1.67)	0.026
PTPRD	0.56 (0.42-0.75)	<0.001
PI3K signaling	0.77 (0.62-0.96)	0.022
HRR signaling	0.77 (0.63-0.95)	0.016

		Mutational event 1		Mutational event 2		Interaction effect	
Mutational event 1	Mutational event 2	HR (95% CI)	P value	HR (95% CI)	P value	HR (95% CI)	P value
EGFR	PI3K signaling	2.06 (1.54-2.76)	<0.001	0.84 (0.66-1.07)	0.16	0.43 (0.23-0.80)	0.008
TP53	*KRAS*	1.04 (0.86-1.27)	0.69	1.10 (0.84-1.43)	0.49	0.51 (0.32-0.80)	0.003
TP53	*ERBB4*	0.98 (0.82-1.17)	0.80	1.58 (0.86-2.90)	0.14	0.36 (0.17-0.79)	0.010
Cell cycle signaling	HRR signaling	1.97 (1.41-2.75)	<0.001	0.91 (0.73-1.14)	0.43	0.26 (0.14-0.49)	<0.001
PI3K signaling	Chromatin remodeling	0.96 (0.74-1.25)	0.76	0.98 (0.79-1.21)	0.83	0.59 (0.37-0.94)	0.026

Abbreviations: CI=confidence interval, HR=hazard ratio, NSCLC=non-small cell lung cancer, PFS=progression-free survival.

Supplementary Table 8. Three prediction models based on the PFS on anti-PD-(L)1 monotherapy of the non-squamous NSCLCs in the training sets.

	Variable	B (coefficient)	HR (95% CI)	P value
Uni-model	*EGFR*	0.476	1.61 (1.24-2.09)	<0.001
	STK11	0.292	1.34 (1.05-1.70)	0.018
	PTPRD	-0.475	0.62 (0.46-0.85)	0.002
	PI3K pathway	-0.234	0.79 (0.63-0.99)	0.043
	HRR pathway	-0.151	0.86 (0.69-1.06)	0.165
Inter-model	*EGFR*	0.635	1.89 (1.40-2.55)	<0.001
	STK11	0.270	1.31 (1.01-1.70)	0.043
	PTPRD	-0.413	0.66 (0.48-0.90)	0.010
	PI3K pathway	0.158	1.17 (0.88-1.56)	0.285
	HRR pathway	-0.024	0.98 (0.78-1.23)	0.838
	TP53	0.092	1.10 (0.89-1.35)	0.395
	KRAS	0.099	1.10 (0.83-1.46)	0.490
	ERBB4	0.445	1.56 (0.84-2.89)	0.157
	Cell cycle pathway	0.599	1.82 (1.29-2.57)	<0.001
	Chromatin remodeling pathway	-0.028	0.97 (0.77-1.22)	0.812
	*EGFR**PI3K pathway	-0.915	0.40 (0.21-0.75)	0.005
	*TP53*KRAS*	-0.554	0.57 (0.36-0.93)	0.024
	*TP53*ERBB4*	-0.735	0.48 (0.22-1.07)	0.071
	HRR pathway*Cell cycle pathway	-1.113	0.33 (0.17-0.62)	<0.001
	PI3K pathway*Chromatin remodeling pathway	-0.474	0.62 (0.39-1.00)	0.052
Null-inter-model	*EGFR*	0.444	1.56 (1.19-2.04)	0.001
	STK11	0.350	1.42 (1.10-1.83)	0.006
	PTPRD	-0.448	0.64 (0.47-0.87)	0.005
	PI3K pathway	-0.205	0.82 (0.65-1.03)	0.081
	HRR pathway	-0.157	0.85 (0.69-1.07)	0.162
	TP53	-0.038	0.96 (0.80-1.16)	0.686
	KRAS	-0.148	0.86 (0.69-1.07)	0.184
	ERBB4	-0.171	0.84 (0.57-1.24)	0.384
	Cell cycle pathway	0.163	1.18 (0.88-1.58)	0.279
	Chromatin remodeling pathway	-0.141	0.87 (0.71-1.07)	0.176

Abbreviations: CI=confidence interval, HR=hazard ratio, NSCLC=non-small cell lung cancer, PD-1=programmed death-1, PD-L1=programmed death-ligand 1, PFS=progression-free survival.

Supplementary Table 9. Univariable-significant single mutational events and significant interactions based on the PFS data of the squamous NSCLCs in the training sets.

	Mutational event 1	
Mutational event 1	HR (95% CI)	P value
NOTCH1/2/3	0.65 (0.41-1.00)	0.053
LRP1B	1.56 (1.09-2.24)	0.016
RB1	1.77 (1.00-3.15)	0.051
PI3K signaling	0.75 (0.53-1.05)	0.092

		Mutational event 1		Mutational event 2		Interaction effect	
Mutational event 1	Mutational event 2	HR (95% CI)	P value	HR (95% CI)	P value	HR (95% CI)	P value
TP53	*NFE2L2*	1.37 (0.97-1.92)	0.071	3.71 (1.15-12.00)	0.029	0.21 (0.06-0.74)	0.016
TP53	*HRR*	1.43 (0.99-2.06)	0.055	1.31 (0.70-2.43)	0.40	0.55 (0.25-1.19)	0.13

Abbreviations: CI=confidence interval, HR=hazard ratio, NSCLC=non-small cell lung cancer, PFS=progression-free survival.

Supplementary Table 10. Three prediction models based on the PFS on anti-PD-(L)1 monotherapy of the squamous NSCLCs in the training sets.

	Variable	B (coefficient)	HR (95% CI)	P value
Uni-model	*NOTCH1/2/3*	-0.398	0.67 (0.43-1.05)	0.080
	LRP1B	0.493	1.64 (1.14-2.36)	0.008
	RB1	0.482	1.62 (0.91-2.89)	0.102
	PI3K pathway	-0.310	0.73 (0.52-1.04)	0.078
Inter-model	*NOTCH1/2/3*	-0.322	0.72 (0.46-1.14)	0.165
	LRP1B	0.521	1.68 (1.14-2.49)	0.009
	RB1	0.543	1.72 (0.95-3.12)	0.074
	PI3K pathway	-0.251	0.78 (0.55-1.11)	0.163
	TP53	0.352	1.42 (0.98-2.07)	0.067
	NFE2L2	0.913	2.49 (0.69-8.96)	0.162
	HRR pathway	0.206	1.23 (0.63-2.38)	0.542
	TP53*NFE2L2	-1.210	0.30 (0.08-1.16)	0.082
	TP53*HRR pathway	-0.509	0.60 (0.26-1.39)	0.235
Null-inter-model	*NOTCH1/2/3*	-0.362	0.70 (0.44-1.09)	0.117
	LRP1B	0.504	1.66 (1.13-2.43)	0.010
	RB1	0.471	1.60 (0.89-2.89)	0.118
	PI3K pathway	-0.308	0.74 (0.52-1.04)	0.085
	TP53	0.200	1.22 (0.87-1.72)	0.248
	NFE2L2	-0.184	0.83 (0.52-1.34)	0.447
	HRR pathway	-0.089	0.91 (0.62-1.35)	0.657

Abbreviations: CI=confidence interval, HR=hazard ratio, NSCLC=non-small cell lung cancer, PD-1=programmed death-1, PD-L1=programmed death-ligand 1, PFS=progression-free survival.

References

1 Chen, J. *et al.* Genomic landscape of lung adenocarcinoma in East Asians. *Nat Genet* **52**, 177-186, doi:10.1038/s41588-019-0569-6 (2020).
2 Xu, J. Y. *et al.* Integrative Proteomic Characterization of Human Lung Adenocarcinoma. *Cell* **182**, 245-261 e217, doi:10.1016/j.cell.2020.05.043 (2020).
3 Gillette, M. A. *et al.* Proteogenomic Characterization Reveals Therapeutic Vulnerabilities in Lung Adenocarcinoma. *Cell* **182**, 200-225 e235, doi:10.1016/j.cell.2020.06.013 (2020).
4 Schoenfeld, A. J. *et al.* Clinical and molecular correlates of PD-L1 expression in patients with lung adenocarcinomas. *Ann Oncol*, doi:10.1016/j.annonc.2020.01.065 (2020).
5 Wang, Z. *et al.* Assessment of Blood Tumor Mutational Burden as a Potential Biomarker for Immunotherapy in Patients With Non-Small Cell Lung Cancer With Use of a Next-Generation Sequencing Cancer Gene Panel. *JAMA Oncol*, doi:10.1001/jamaoncol.2018.7098 (2019).
6 Gandara, D. R. *et al.* Blood-based tumor mutational burden as a predictor of clinical benefit in non-small-cell lung cancer patients treated with atezolizumab. *Nat Med* **24**, 1441-1448, doi:10.1038/s41591-018-0134-3 (2018).
7 Rizvi, H. *et al.* Molecular Determinants of Response to Anti-Programmed Cell Death (PD)-1 and Anti-Programmed Death-Ligand 1 (PD-L1) Blockade in Patients With Non-Small-Cell Lung Cancer Profiled With Targeted Next-Generation Sequencing. *J Clin Oncol* **36**, 633-641, doi:10.1200/JCO.2017.75.3384 (2018).
8 Munari, E. *et al.* PD-L1 expression in non-small cell lung cancer: evaluation of the diagnostic accuracy of a laboratory-developed test using clone E1L3N in comparison with 22C3 and SP263 assays. *Hum Pathol* **90**, 54-59, doi:10.1016/j.humpath.2019.05.003 (2019).
9 Adam, J. *et al.* Multicenter harmonization study for PD-L1 IHC testing in non-small-cell lung cancer. *Ann Oncol* **29**, 953-958, doi:10.1093/annonc/mdy014 (2018).
10 Newman, A. M. *et al.* Robust enumeration of cell subsets from tissue expression profiles. *Nat Methods* **12**, 453-457, doi:10.1038/nmeth.3337 (2015).
11 Rizvi, N. A. *et al.* Cancer immunology. Mutational landscape determines sensitivity to PD-1 blockade in non-small cell lung cancer. *Science* **348**, 124-128, doi:10.1126/science.aaa1348 (2015).
12 Hellmann, M. D. *et al.* Genomic Features of Response to Combination Immunotherapy in Patients with Advanced Non-Small-Cell Lung Cancer. *Cancer Cell* **33**, 843-852 e844, doi:10.1016/j.ccell.2018.03.018 (2018).
13 Fang, W. *et al.* Comprehensive Genomic Profiling Identifies Novel Genetic Predictors of Response to Anti-PD-(L)1 Therapies in Non-Small Cell Lung Cancer. *Clin Cancer Res* **25**, 5015-5026, doi:10.1158/1078-0432.CCR-19-0585 (2019).
14 Miao, D. *et al.* Genomic correlates of response to immune checkpoint blockade in microsatellite-stable solid tumors. *Nat Genet* **50**, 1271-1281, doi:10.1038/s41588-018-0200-2 (2018).
15 Samstein, R. M. *et al.* Tumor mutational load predicts survival after immunotherapy across multiple cancer types. *Nat Genet* **51**, 202-206, doi:10.1038/s41588-018-0312-8 (2019).
