# Supplementary material for: Co-occurring genomic alterations and immunotherapy efficacy in NSCLC
Source: NPJ Precis Oncol. 2022 Jan 18;6:4. doi: 10.1038/s41698-021-00243-7 (PMC8766442; doi:10.1038/s41698-021-00243-7)
Supplement: Supplementary file 2 — Reporting Summary [file 41698_2021_243_MOESM2_ESM.pdf]

nature portfolio

Corresponding author(s): Lei Zhao and Yi Hu

Last updated by author(s): Aug 25, 2021

Reporting Summary

Nature Portfolio wishes to improve the reproducibility of the work that we publish. This form provides structure for consistency and transparency in reporting. For further information on Nature Portfolio policies, see our Editorial Policies and the Editorial Policy Checklist.

Statistics

For all statistical analyses, confirm that the following items are present in the figure legend, table legend, main text, or Methods section.

n/a	Confirmed	
☐	☒	The exact sample size (*n*) for each experimental group/condition, given as a discrete number and unit of measurement
☐	☒	A statement on whether measurements were taken from distinct samples or whether the same sample was measured repeatedly
☐	☒	The statistical test(s) used AND whether they are one- or two-sided<br>*Only common tests should be described solely by name; describe more complex techniques in the Methods section.*
☐	☒	A description of all covariates tested
☐	☒	A description of any assumptions or corrections, such as tests of normality and adjustment for multiple comparisons
☐	☒	A full description of the statistical parameters including central tendency (e.g. means) or other basic estimates (e.g. regression coefficient) AND variation (e.g. standard deviation) or associated estimates of uncertainty (e.g. confidence intervals)
☐	☒	For null hypothesis testing, the test statistic (e.g. *F*, *t*, *r*) with confidence intervals, effect sizes, degrees of freedom and *P* value noted<br>*Give P values as exact values whenever suitable.*
☒	☐	For Bayesian analysis, information on the choice of priors and Markov chain Monte Carlo settings
☒	☐	For hierarchical and complex designs, identification of the appropriate level for tests and full reporting of outcomes
☐	☒	Estimates of effect sizes (e.g. Cohen's *d*, Pearson's *r*), indicating how they were calculated

Our web collection on statistics for biologists contains articles on many of the points above.

Software and code

Policy information about availability of computer code

Data collection	N.A.
Data analysis	We used IBM SPSS Statistics 25 and R version 4.1.0 for data analysis. The packages we performed in R include "ggplot2", "ggpubr", "magrittr", "survival", "survminer", "reshape2", "forcats", "rms", "foreign", "caret", "PresenceAbsence", "riskRegression", and "prodlim".

For manuscripts utilizing custom algorithms or software that are central to the research but not yet described in published literature, software must be made available to editors and reviewers. We strongly encourage code deposition in a community repository (e.g. GitHub). See the Nature Portfolio guidelines for submitting code & software for further information.

Data

Policy information about availability of data

All manuscripts must include a data availability statement. This statement should provide the following information, where applicable:

- Accession codes, unique identifiers, or web links for publicly available datasets
- A description of any restrictions on data availability
- For clinical datasets or third party data, please ensure that the statement adheres to our policy

The authors declare that relevant data supporting the findings of this study are available within the paper and its Supplementary files. TThe source of the data (hyperlinks and DOIs) of all the included datasets are shown in Supplementary Table 1. The data of the NCC and SYSUCC cohorts are obtained through sending requests to the corresponding authors. Due to ethical and privacy concerns, we are sorry that we are unable to publish their full data in our study. Readers may contact the corresponding authors for the access of individual patient-level data for non-commercial purposes.

Field-specific reporting

Please select the one below that is the best fit for your research. If you are not sure, read the appropriate sections before making your selection.

☒ Life sciences ☐ Behavioural & social sciences ☐ Ecological, evolutionary & environmental sciences

For a reference copy of the document with all sections, see nature.com/documents/nr-reporting-summary-flat.pdf

Life sciences study design

All studies must disclose on these points even when the disclosure is negative.

Sample size	We analyzed the clinical data of all published cohorts of NSCLC patients who had received immunotherapy. The sample size is sufficient for our analysis of the interaction effects between different mutational events.
Data exclusions	We excluded the replicated data among the included cohorts by patient identifier (e.g., P-0003869).
Replication	We employed two training sets for cross-validation and two validation sets for independent validation.
Randomization	No randomization was performed. We implemented subgroup analysis and multivariable analysis to exclude the potential influence from covariates.
Blinding	Blinding is irrelevant to the present study.

Reporting for specific materials, systems and methods

We require information from authors about some types of materials, experimental systems and methods used in many studies. Here, indicate whether each material, system or method listed is relevant to your study. If you are not sure if a list item applies to your research, read the appropriate section before selecting a response.

Materials & experimental systems

n/a	Involved in the study	
☒	☐	Antibodies
☒	☐	Eukaryotic cell lines
☒	☐	Palaeontology and archaeology
☒	☐	Animals and other organisms
☐	☒	Human research participants
☒	☐	Clinical data
☒	☐	Dual use research of concern

Methods

n/a	Involved in the study	
☒	☐	ChIP-seq
☒	☐	Flow cytometry
☒	☐	MRI-based neuroimaging

Human research participants

Policy information about studies involving human research participants

Population characteristics	Eight cohorts of 1,745 NSCLC patients were analyzed, from National Cancer Center (NCC), SYSUCC, DFCI, MSKCC, the POPLAR/OAK trial, and TCGA database. The characteristics (sex, age, race, smoking status, ECOG, metastatic status, TMB, PD-L1, and ICI usage) of the included patients who had received immunotherapy are shown in Fig. 3M and 5O. The characteristics of cohorts are accessible online (PMID: 25765070, 30082870, 30643254, 30150660, 30816954, 29337640, 31085721, 29657128).
Recruitment	No recruitment was performed in the present study. We analyzed the clinical data of all published cohorts of NSCLC patients who had received immunotherapy. The recruitment criteria are accessible online (PMID: 25765070, 30082870, 30643254, 30150660, 30816954, 29337640, 31085721, 29657128).
Ethics oversight	Institution Review Board of Chinese PLA General Hospital (2015L01380).

Note that full information on the approval of the study protocol must also be provided in the manuscript.
